# Strategic Mobility Engineering in 2D Semiconductor‐based FETs for Enhanced Electronic Devices

**DOI:** 10.1002/advs.202509170

**Published:** 2025-09-17

**Authors:** Sheila Sim, Sichao Li, Weifan Cai, Baiyu Yu, Xin Ju, Jing Cao, Zhaogang Dong, Xinmao Yin, Sunmi Shin, Yue Luo, Dongzhi Chi, Ady Suwardi, Hong Kuan Ng, Jing Wu

**Affiliations:** ^1^ School of Electronic Science & Engineering Southeast University Nanjing 211189 China; ^2^ Institute of Materials Research and Engineering (IMRE), Agency for Science Technology and Research (A*STAR) 2 Fusionopolis Way, Innovis #08‐03 Singapore 138634 Republic of Singapore; ^3^ Department of Mechanical Engineering College of Design and Engineering National University of Singapore Singapore 117575 Singapore; ^4^ Science, Mathematics, and Technology (SMT) Singapore University of Technology and Design (SUTD) 8 Somapah Road Singapore 487372 Singapore; ^5^ Shanghai Key Laboratory of High Temperature Superconductors Institute for Quantum Science and Technology, Physics Department Shanghai University Shanghai 200444 China; ^6^ Department of Electronic Engineering The Chinese University of Hong Kong Sha Tin, New Territories Hong Kong SAR 999077 China

**Keywords:** 2D materials, field‐effect‐transistor, mobility engineering

## Abstract

As silicon‐based electronics approach the physical limits of Moore's Law, 2‐Dimensional (2D) semiconductors emerge as promising candidates for next‐generation electronic devices due to their atomic‐scale thickness and inherently high carrier mobilities. These materials offer superior electrostatic control, mitigating short‐channel effects while enabling continued device scaling. However, challenges such as contact resistance and suboptimal channel properties continue to impede carrier transport, necessitating advanced mobility engineering strategies. This review comprehensively evaluates recent approaches to enhance carrier mobility in 2D semiconductor‐based field‐effect transistors (FETs), including doping, metal‐semiconductor interface optimization, effective mass engineering, scattering mechanism manipulation, work function tuning, and strain engineering. These strategies improve critical device parameters like current drive, subthreshold swing, and on/off ratios by optimizing carrier transport efficiency. By linking material‐level advancements to circuit‐level performance, this work underscores the pivotal role of mobility engineering in enabling scalable, high‐performance 2D electronics. These insights pave the way for transitioning 2D materials from laboratory research to practical applications, overcoming the limitations of conventional silicon technologies and driving innovations in high‐performance, energy‐efficient electronics.

## Graphical abstract

This review highlights device‐level strategies to optimize carrier transport in 2D‐FETs, focusing on channel engineering and contact interface design. Techniques such as doping, strain tuning, and contact resistance reduction are discussed to improve mobility and drive current. By bridging material innovation and device performance, it outlines pathways toward scalable, high‐performance 2D semiconductor electronics.

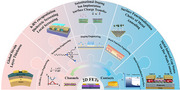

## 1 Introduction

The debate over whether Moore's Law is still viable or has reached its end remains contentious. For decades, the semiconductor industry has sustained the pace of Moore's Law through continuous innovation in device architecture, material engineering, and fabrication technologies. Despite extensive global efforts in miniaturization and integration, many have predicted the demise of Moore's Law owing to fundamental physical limitations and manufacturing challenges that impede further advancement.^[^ ^1^, ^2^ ^]^ As device dimensions approach the atomic scale, new challenges related to performance and reliability have emerged, particularly concerning the fundamental limits of dimensional scaling. Early efforts focused primarily on shrinking transistor dimensions to sustain the trajectory of Moore's Law. While this strategy has been effective for decades, continued scaling now encounters physical and material limitations that require alternative approaches to device innovation and thermal management. Innovations such as strained silicon, high‐k dielectric, and metal gate, FinFET, and gate‐all‐around FETs (GAAFET) technologies have successfully scaled transistors to the nanoscale domain.^[^ ^3^, ^4^, ^5^ ^]^ However, these innovations have only temporarily mitigated inherent issues like short‐channel effects, heat dissipation, and surface degradation, all of which degrade carrier mobility and increase performance variability to adversely affect performance. This has spurred the exploration of alternative materials to meet the industry's demand for faster, smaller, and more efficient devices.^[^ ^6^ ^]^

Among the leading candidates for beyond‐silicon electronics are 2D semiconductors, which exhibit exceptional electrical, mechanical, and thermal properties, along with atomic‐scale thicknesses that enable superior electrostatic control. Since their discovery in 2004, 2D materials have undergone tremendous advancements and have been highlighted in the International Roadmap for Devices and Systems (IRDS) for their potential to revolutionize semiconductor electronic devices and quantum‐engineered devices and their compatibility with heterogeneous integration into existing silicon technologies.^[^ ^7^ ^]^ Unlike conventional semiconductors such as silicon (Si) and gallium arsenide (GaAs), whose carrier mobilities drop drastically when thinned down to a nanometer (≈1−10 cm^2^ V^−1^ s^−1^),^[^ ^8^ ^]^ many 2D semiconductors retain notable mobilities (≈100 cm^2^ V^−1^ s^−1^) even as single atomic layers.^[^ ^9^, ^10^ ^]^ Carrier mobility is a fundamental parameter that determines the speed and efficiency of electronic devices, as it quantifies how efficiently charge carriers ‐ electrons or holes ‐ can move through a semiconductor material in response to an applied electric field. High carrier mobility enables faster switching speeds, lower power consumption, and overall improved device performance, making it a critical factor in the design and selection of materials for next‐generation electronics.

In 2D semiconductors, mobility is strongly influenced by three key parameters: operating temperature, carrier concentration, and defects/impurities. First, the operating temperature determines the type of dominant scattering phenomenon within the semiconductor.^[^ ^9^ ^]^ For instance, in typical 2D semiconductors like transition metal dichalcogenides (TMDCs), electron‐phonon scattering is the primary factor limiting carrier mobility at room‐temperature. Taking MoS_2_ as an example, its theoretical phonon‐limited mobility can only reach up to 410 cm^2^ V^−1^ s^−1^ at room‐temperature.^[^ ^9^, ^10^, ^11^ ^]^ Second, the effect of carrier concentration on mobility is often underestimated. At low concentrations (<10^13^ cm^−2^), scattering due to intrinsic and extrinsic defects and impurities becomes dominant, decreasing mobility.^[^ ^9^ ^]^ At high concentrations (>10^13^ cm^−2^), although beneficial for screening defect/impurity potential, coulombic interactions between carriers and high‐energy scattering centers intensify, leading to reduced mobility.^[^ ^11^ ^]^ Lastly, interfacial defects such as wrinkles or substrate inhomogeneities arising from lattice mismatch or chemical impurities can severely affect scattering mechanisms and lead to fluctuations in carrier concentration and mobility.^[^ ^12^ ^]^

Given the central role of transistors in electronic applications, the electrical characteristics of 2D materials are often evaluated using the FET platform.^[^ ^13^ ^]^ A typical FET is a three‐terminal electronic device consisting of source, drain, and gate electrodes. In operation, a voltage applied to the gate electrode modulates the density of charge carriers ‐ electrons or holes ‐ in a semiconductor channel positioned between the source and drain. This modulation controls the current flow between source and drain terminals, making FETs essential for digital logic and amplification applications in electronics. The key performance metrics include the carrier mobility, ON/OFF ratio, leakage current and subthreshold swing (SS).^[^ ^14^ ^]^ In recent years, 2D semiconductors utilizing FET structures have demonstrated substantial progress, with several exhibiting superior mobility characteristics compared to their bulk counterparts. For instance, bilayer MoS_2_ demonstrated a leap in room‐temperature mobility reaching values ≈900 cm^2^ V^−1^ s^−1^, far exceeding theoretical predictions.^[^ ^10^ ^]^ Few‐layer InSe (three layers ≈2.4 nm) has demonstrated ultra‐high electron mobility over 1000 cm^2^ V^−1^ s^−1^, although this value decreases with increasing monolayer thicknesses.^[^ ^15^, ^16^ ^]^ Conversely, materials like Bi_2_O_2_Se, which possess similar band gap and electron mobility as Si (0.85 eV and 3100 cm^2^ V^−1^ s^−1^)^[^ ^17^, ^18^ ^]^, maintain performance metrics even at the monolayer.^[^ ^19^ ^]^ WSe_2_ shows high room‐temperature hole mobility of over 1000 cm^2^ V^−1^ s^−1^ owing to charge transfer doping,^[^ ^20^ ^]^ meeting the IRDS requirement for nodes beyond 3 nm.^[^ ^21^ ^]^ These properties, combined with the unique electronic^[^ ^21^ ^]^ and tunable bandgap^[^ ^22^ ^]^ characteristics of other 2D materials such as graphene, TMDCs, Black Phosphorous (BP) and more underscore the potential of 2D semiconductors in future electronics.

Despite their promise, 2D materials face major hurdles that hinder their transition and adoption in practical electronics. Challenges such as high contact resistance, poor crystal quality, and incompatible dielectric environments degrade carrier mobility. Additionally, intrinsic factors such as strong phonon‐related scattering, surface roughness, and impurities restrict performance at room temperature, resulting in suboptimal carrier mobilities.^[^ ^8^ ^]^ To address these limitations, numerous research efforts have been invested in seeking strategies for improving carrier mobility, aiming to not only surpass theoretical limits in 2D semiconductors but also unlock new possibilities for high‐performance 2D electronics.

**Figure 1:**
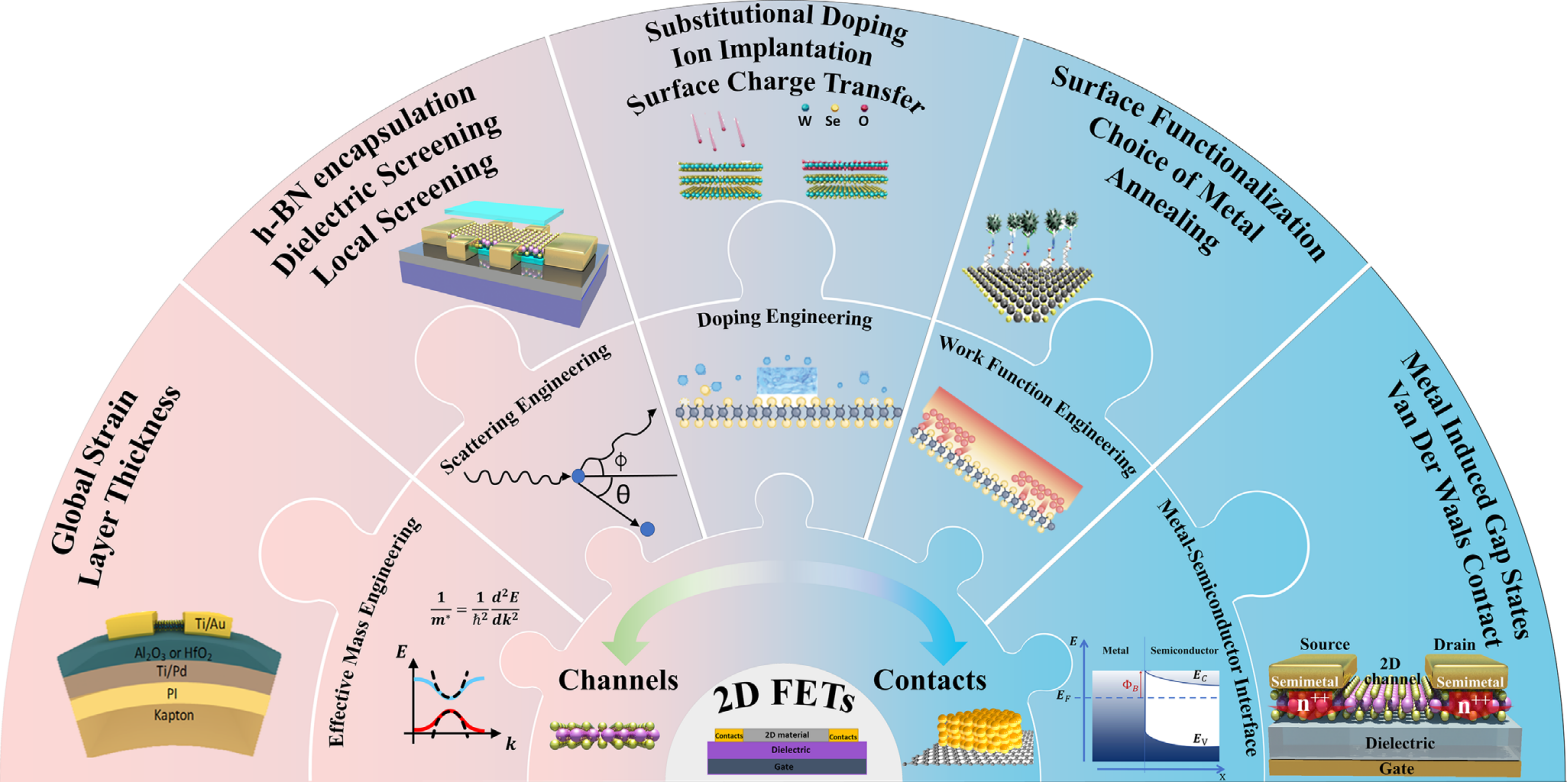
Schematic overview of the 2D FET platform, highlighting key approaches and parameters for mobility engineering through channel and contact optimization.^[^ ^85^, ^124^, ^125^ ^]^

This review provides a comprehensive evaluation of recent mobility engineering strategies aimed at enhancing the performance of 2D semiconductor FETs. **Figure** **1** illustrates the intricate relationships within the 2D FET platform, highlighting key strategies explored to enhance mobility, including doping effect, metal‐semiconductor interface optimization, effective mass engineering, scattering mechanism manipulation, work function tuning and strain engineering. Collectively, these strategies provide valuable insights into overcoming longstanding challenges and enhancing carrier mobility in 2D devices, which play a crucial role in bridging the gap between laboratory‐scale research and the development of practical, high‐performance electronic systems, paving the way for technologies that surpass the limitations of traditional silicon‐based platforms.

## 2 Main Challenges in Achieving High Mobility 2D FETs

In the field of 2D electronics and their diverse applications, the transistor serves as the fundamental building block. Among various transistor designs, field‐effect transistors (FETs) hold particular significance, as they provide an essential and direct platform for examining the electrical properties of atomically thin semiconductors and evaluating their performance in functional devices. A typical 2D FET comprises a semiconductor material as the channel, with metallic drain and source electrodes at either end, and a dielectric layer that serves as a gate to modulate carrier concentration.^[^ ^14^ ^]^ Depending on the applied drain‐source voltage (*V_ds_*) and gate voltage (*V_gs_*), the carrier mobility (*µ*) of 2D FETs can be tuned.^[^ ^13^ ^]^

According to the Shockley model,^[^ ^21^ ^]^ the source‐drain current of an ideal FET in the linear region is expressed as follows:

(1) $$I_{ds}=\mu_{eff\;}C\frac{W}{L}\left[\left(V_{gs}-V_{t}\right)V_{ds}-\frac{V_{ds}^{2}}{2}\right]$$

(2) $$\mu_{FE}=\frac{dI_{ds}}{dV_{g}}\frac{1}{V_{ds}}\frac{L}{W}$$

Here, µ_*eff*_, µ_*FE*_, *W*, *L*, *C*, *V_gs_*, *V_t_* and *I_ds_*, represent the effective mobility, FET mobility, channel width, channel length, gate oxide capacitance, gate‐source voltage, threshold voltage, drain‐source current of the 2D semiconductors channel, respectively.^[^ ^13^, ^21^ ^]^

**Figure 2:**
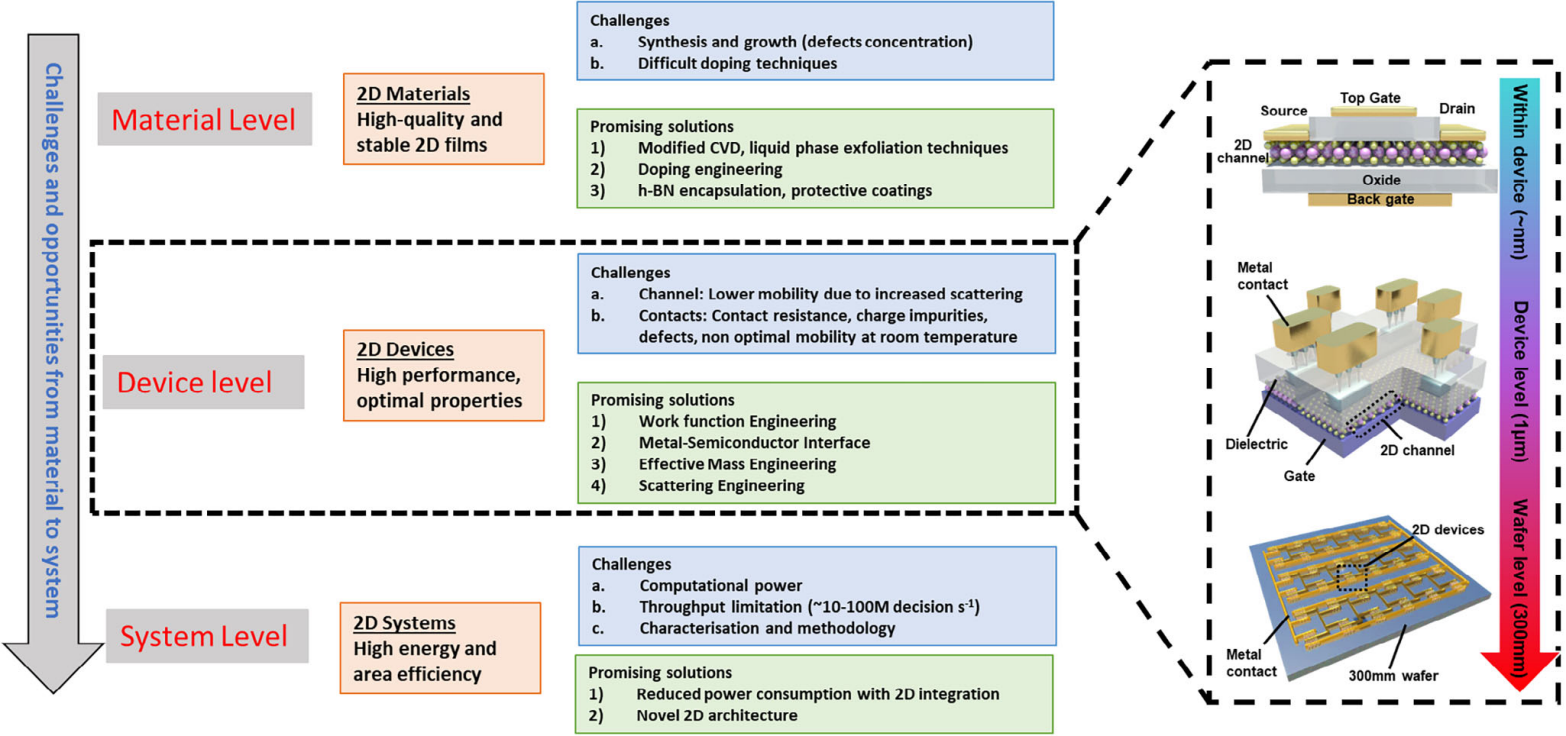
Roadmap outlining the integration of 2D materials for next‐generation computing. The roadmap highlights the challenges and potential solutions in three perspectives: material, device and system.^[^ ^109^ ^]^ Right: scaling characteristics of transistors integrated on a 300mm CMOS line.

To realize highly efficient 2D devices with ultra‐low power consumption for advanced technological applications,^[^ ^23^ ^]^ several critical challenges must be systematically addressed across the material, device, and system levels (**Figure** **2**), each presenting distinct challenges as well as unique opportunities. At the material level, preserving atomic‐scale structural integrity and minimizing defects are critical for enhancing mobility. Device‐level challenges arise from the implications brought forth by the channel, contacts, and their interface. System‐level challenges involve scalable integration of 2D materials into reliable architectures that meet the demands of electronics manufacturing.

Within this multilevel framework, carrier mobility emerges as the most direct figure‐of‐merit for evaluating charge‐transport efficiency, yet it is governed by two fundamentally different scales of influence. At the materials‐level, the goal is to select or design crystals whose intrinsic mobility (µ_*int*_) is already high, as µ_*int*_ represents the theoretical upper limit of carrier transport within a perfect crystal, constrained solely by fundamental interactions such as phonons scattering, band structure, intrinsic dielectric screening etc.^[^ ^24^, ^25^, ^26^, ^27^, ^28^ ^]^ For example, the µ_*int*_ of monolayer MoS_2_ at room temperature is theoretically predicted to be ≈410 cm^2^ V^−1^ s^−1^, limited primarily by optical phonons scattering.^[^ ^29^ ^]^ Improving µ_*int*_ requires atomic‐scale engineering strategies such as phonon dispersion modulation, strain tuning, and defect passivation. Experimentally, however, µ_*int*_ is challenging to measure due to unavoidable extrinsic contributions to scattering such as contact resistance, dielectric environment, and interface trap states. In contrast, the field‐effect mobility (µ_*FE*_) can be extracted from transfer characteristics of a transistor at the device‐level, reflecting not only intrinsic scattering but also extrinsic penalties mentioned above. As such, µ_*FE*_ provides the most realistic benchmark for gauging engineering progress and device‐level transport performance. Notably, phonon‐limited transport has been observed in monolayer MoS_2_ through hexagonal boron nitride (h‐BN) encapsulation and dielectric engineering, achieving µ_*FE*_ values approaching ≈200 cm^2^ V^−1^ s^−1^ at room temperature‐roughly half of the theoretical ceiling.^[^ ^30^, ^31^ ^]^ Closing this gap is therefore less a question of discovering new materials than of perfecting contact quality, dielectric interfaces, and fabrication processes‐precisely the focus of the mobility‐engineering strategies reviewed in the following sections.

Recent years have seen particular progress in the advancement of 2D materials and it is important to note that this review does not aim to provide an exhaustive overview of the challenges and opportunities presented in the broad field of 2D materials and devices. Instead, we focus on mobility engineering strategies and the developments made at the device level and emphasize the critical roles of contact and interface optimization. By doing so, we aim to inspire further innovations and expansion of the current methodologies toward mobility engineering of 2D devices and highlight the significance of continued efforts to unlock the full potential of 2D materials in electronics.

### 2.1 Channel

The FET channel is a narrow region situated between the source and drain electrodes, serving as the pathway for charge carriers. These carriers move through the channel under the influence of an applied electric field, modulated by the gate voltage, which controls the carrier concentration and thus the current flow in the device.^[^ ^21^ ^]^ Typically composed of a semiconductor material such as silicon, the channel is intentionally doped to form either an *n*‐type or *p*‐type region, depending on the desired operation of the device. In an *n*‐type channel, electrons serve as the majority carriers, whereas in a *p*‐type channel, holes dominate. The critical factors that influence carrier mobility include the channel thickness, doping concentration, and the presence of various scattering mechanisms. Thinner channels can enhance electrostatic control but also leads to increase of surface roughness and phonon scattering. Similarly, higher doping levels can improve the carrier concentration but may introduce ionized impurity scattering.^[^ ^32^ ^]^ Understanding and optimizing these factors are essential for maximizing carrier mobility and overall device performance, offering a degree of freedom to engineer carrier mobility in 2D semiconductors, which will be further discussed in the following sections.

### 2.2 Contacts

Metal–semiconductor (M–S) contacts are essential components in any transistor, as they govern the efficiency of charge injection between the external circuit and the semiconductor channel. Over the past few decades, these contacts on bulk semiconductors have been optimized by heavily doping the contact region to decrease the space charge width.^[^ ^33^ ^]^ This enables efficient electron or hole tunnelling to achieve efficient carrier transport and minimize resistance. However, achieving ohmic contacts on atomically thin 2D semiconductors remains a significant challenge, primarily due to the difficulty in effectively doping these materials and the potential for damage during the fabrication process. These issues can lead to high contact resistance, which in turn hampers charge injection and degrades the overall performance of electronic devices.

It has been reported that doping 2D semiconductors can destabilize their structure and introduce defects.^[^ ^8^ ^]^ The space charge width in 2D semiconductors is only weakly dependent on doping concentration, making traditional methods for achieving *p*‐type and *n*‐type devices unsuitable. An alternative approach involves modifying the Schottky barrier height at the metal‐semiconductor (M‐S) contacts by carefully selecting contact metals with work functions that align favorably with the band edges of the 2D semiconductor. This strategy can facilitate more efficient carrier injection, reduce contact resistance, and improve overall device performance without the need for conventional doping. However, depositing metals on atomically thin materials introduces defects resulting from physical damage during deposition or chemical reactions with metal atoms, intrinsic defects or adsorbed contaminants on the 2D semiconductor surface.^[^ ^34^ ^]^ These defects at the interface can cause Fermi level pinning, resulting in large Schottky barriers that cannot be modulated.

## 3 Mobility Engineering Strategies

Recent progress in fabrication techniques and innovative approaches in theoretical and experimental studies have allowed substantial improvements in mobility by tailoring the crystal structure, strain, and defects in 2D materials.^[^ ^8^ ^]^ These advancements have opened promising opportunities for developing next‐generation electronic devices with high carrier mobility at room temperature. The effectiveness of each mobility engineering strategy is highly material dependent and selecting the appropriate strategy requires considering the intrinsic properties of the intended application. **Table** **1** summarizes the advantages and limitations of each mobility engineering approach, and the following sections will introduce the latest developments and innovations of each approach, highlighting their potential to enhance carrier mobility and device performance.

**Table 1:** Comparative analysis of mobility engineering approaches for 2D FETs. The table highlights the benefits and challenges associated with each approach, providing a detailed overview of the trade‐offs and opportunities.

Engineering approach	Key advantages	Key limitations
Effective Mass Engineering	• Can be achieved by adjusting layer thickness • Can be achieved by applying strain • Enables band‐structure optimization for carriers	• Mobility vs effective mass relationship is material‐dependent • Applying uniform strain in devices is experimentally challenging
Scattering Engineering	• Can suppress dominant scattering mechanisms (e.g., phonons or impurities) to enhance mobility • Tailors charge transport to desired regime	• Some scattering sources (e.g., phonons) cannot be completely eliminated • Dependent on external environment and fabrication processes
Doping Engineering	• Directly modulates carrier concentration in 2D channel • Allows creation of p–n junctions and contact doping for injection improvement	• Dopants can introduce defects or traps that counteract mobility gains • Precise control over dopant placement and density is difficult in 2D lattices
Work Function Engineering	• Enables tuning of Schottky barrier height • Improves carrier injection efficiency • Multiple methods available (e.g., surface functionalization, annealing)	• Limited range of achievable work function shifts • Some methods (e.g., chemical functionalization) may have stability/uniformity issues
Metal‐Semiconductor Interface Engineering	• Capable of significantly reducing contact resistance • Approaches like edge or van der Waals (vdW) contacts improve injection and minimize damage	• Achieving ideal ohmic contacts challenging in practice • Some contact engineering methods add process complexity or are not yet wafer‐scale

### 3.1 Metal Semiconductor Interface

Among various mobility engineering strategies, metal‐semiconductor (M‐S) interface optimization directly influences device performance by reducing contact resistance. In 2D semiconductor‐based FETs, high carrier mobility is fundamentally limited by contact resistance (*R_c_*), which governs charge injection efficiency from metal electrodes into the semiconductor channel. Despite the intrinsic channel potentially exhibiting superior transport properties, elevated *R_c_* values can overshadow these capabilities by dominating overall device resistance. Thus, optimizing the quality of the M‐S interface is critical, as poor interface conditions result in Schottky barrier formation and Fermi‐level pinning, prominently impeding charge injection and restricting mobility enhancement.^[^ ^35^ ^]^ Particularly for 2D semiconductors, the atomically thin structure is vulnerable to damage during metal deposition, and the absence of dangling bonds reduces orbital overlap with metals, presenting further challenges in achieving low *R_c_*.

Theoretically, in ballistic transport within ultra‐short channel lengths, the minimal achievable contact resistance (*R*_*c*,*min*_) in an ideal M‐S junction is given by:

(3) $$R_{c,min}=\frac{h}{2q^{2}}\sqrt{\frac{\pi }{2n_{2D}}}$$

where *h* is Planck's constant, *q* is the unit charge and *n_2D_* is the carrier concentration in the semiconductor.^[^ ^36^ ^]^ However, achieving this ideal limit is challenging in van der Waals (vdW) materials due to their dangling‐bond‐free surfaces. Key parameters that influence *R_c_* include the Schottky barrier height, tunnel barrier width, and the degree of metal‐semiconductor orbital hybridization.^[^ ^36^, ^37^ ^]^ To overcome this bottleneck, various strategies, including the selection of suitable contact materials, edge contacts, and vdW integration, have thus been pursued to mitigate these challenges and enhance mobility.

#### 3.1.1 Metal Induced Gap States (MIGS)

**Figure 3:**
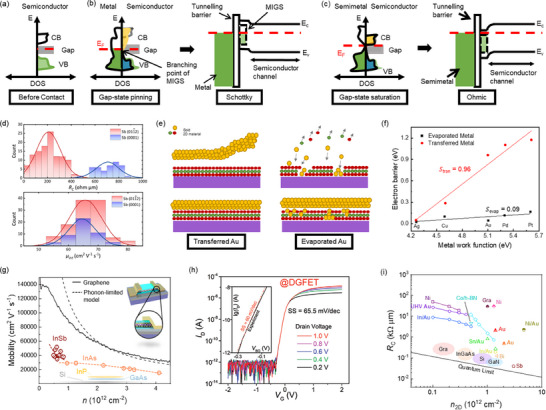
The concept of gap‐state saturation at semimetal–semiconductor interface and vdW contacts. a) The reference DOS of the semiconductor before contact. b) The DOS of normal metal and semiconductor contact. The contributions of the CB and VB to the MIGS are shaded as yellow and blue areas, respectively. Band structure of Schottky barrier due to the gap state pinning c) The DOS of semimetal and semiconductor contact. The Fermi level of the semimetal aligns with the conduction band of the semiconductor, and the DOS at the Fermi level of the semimetal is near zero, conduction band contributed MIGS are suppressed, and the branching point is elevated into the conduction band. Band structure of Ohmic contacts due to gap‐state saturation.^[^ ^38^ ^]^ Reproduced with permission, Copyright 2021 Springer Nature. d) *R*_c_ and intrinsic mobility for Sb(0112) and Sb(0001) red and blue respectively^[^ ^36^ ^]^. Reproduced with permission, Copyright 2023 Springer Nature. e) Cross‐sectional schematics comparing evaporated and transferred Au electrode on top of MoS_2_, showing atomically sharp and clean metal‐semiconductor interfaces versus damaged interface. f) Extraction of threshold voltage (V_th_) on‐state current densities.^[^ ^43^ ^]^ Reproduced with permission, Copyright 2018 Springer Nature. g) Solid black curve in the graph represents the room‐temperature mobility plotted against carrier density. Dashed black curve indicates the theoretical limit of mobility imposed by acoustic‐phonon scattering.^[^ ^50^ ^]^ Reproduced with permission, Copyright 2013 AAAS. Inset: Schematic illustrates the fabrication process for creating edge contacts. h) Transfer curves of WSe_2_‐based PFGFET showing reconfigurability in the FET mode.^[^ ^54^ ^]^ Reproduced with permission, Copyright 2021 Wiley‐VCH GmbH. i) *R*_c_ as a function of *n*_2D_ for monolayer MoS_2_ and other semiconductors in the literature. The black dotted line represents the quantum limit.^[^ ^36^, ^38^ ^]^

The original density of states (DOS) in a semiconductor serves as a baseline for its electronic structure (**Figure** **3**a). Introducing a metal interface can effectively alter the semiconductor's electronic landscape, creating metal‐induced gap states (MIGS) within the semiconductor bandgap, as depicted in Figure 3b. MIGS strongly influence interfacial charge distributions and Schottky barrier heights, subsequently increasing *R_c_* and impeding charge injection efficiency. These MIGS exhibit a DOS profile that reflects the characteristics of the contacting metal. Specifically, they include donor‐like states near the valence band edge and acceptor‐like states near the conduction band edge of the semiconductor.^[^ ^38^ ^]^ This leads to a phenomenon known as gap‐state pinning, where the Fermi level becomes fixed near the intersection of the metal‐induced gap states. Although this pinned state is energetically favorable, it can result in the formation of a Schottky barrier if the Fermi level lies within the semiconductor's bandgap. Such a barrier severely hinders charge injection, thereby limiting the efficiency and performance of the device.

To address MIGS‐induced limitations, research has explored contact materials that minimize these states and reduce *R_c_*. Common metal contacts such as Ti, Au, Cr, and Pd have been extensively studied in MoS_2_‐based devices. Kim et al.^[^ ^39^ ^]^ fabricated transistors using four different metals on a single MoS_2_ flake to compare their electrical behaviors. Their findings revealed that Ti and Au contacts led to devices with the highest quality which exhibited high current levels (up to 10^−4^ A) with ohmic behaviour, while devices fabricated using Cr and Pd contacts displayed low current levels (below 10^−6^ A) and Schottky behaviour. As expected, the devices with lower R_C_ values displayed higher mobility (60‐70 cm^2^ V^−1^ s^−1^). Specifically, Ti contacts showed the lowest R_C_ (32 kΩ∙µm) and highest mobility (70 cm^2^ V^−1^ s^−1^), while Cr contacts exhibited the highest R_C_ (99 kΩ∙µm) and lowest mobility (60 cm^2^ V^−1^ s^−1^).^[^ ^40^, ^41^ ^]^

Semi‐metallic materials like antimony (Sb) and bismuth (Bi) have also been explored for their ability to reduce MIGS. These materials have a nearly zero DOS at the Fermi level, which reduces MIGS and induces a degenerate state, effectively eliminating Schottky barrier. Experimental studies on monolayer MoS_2_ devices have shown that using Sb^[^ ^36^ ^]^ and Bi^[^ ^38^ ^]^ contacts can greatly reduce contact resistance, with Sb contacts achieving 42 Ω µm^−1^ and Bi contacts reaching 123 Ω µm^−1^. As a result of the reduced contact resistance and improved carrier injection, these devices with Sb and Bi contacts exhibit higher mobility of 90 cm^2^ V^−1^ s^−1^ and 120 cm^2^ V^−1^ s^−1^, respectively.

Notably, Bi‐contacted devices demonstrate superior electron mobility with peak values of 120 cm^2^ V^−1^ s^−1^ at 77K and 55 cm^2^ V^−1^ s^−1^ and average value of 30 cm^2^ V^−1^ s^−1^ at room temperature, representing up to three‐orders of magnitude enhancement when compared to Ti (0.03 cm^2^ V^−1^ s^−1^) and Ni contacts (3 cm^2^ V^−1^ s^−1^) in devices with the same aspect ratio, revealing the substantial impact of contacts quality on device performance. However, Bi contacts are susceptible to thermal instability, limiting its applicability due to its low melting point (271.5 °C).^[^ ^38^ ^]^ In contrast, Sb‐contacted devices exhibit excellent electrical performance, stability, and variability, exhibiting *R_c_* values approaching the quantum limit due to sp hybridization. Specifically, Sb ($0\underline{1}12$) contacts exhibited the lowest *R_c_* with on‐state currents ≈1.5 mA µm^−1^ at short channel lengths, outperforming other similar contacts (Figure 3d). Comparisons of crystal orientation highlight Sb ($0\underline{1}12$) as a promising contact material over its Sb (0001) counterpart due to stronger vdW interactions and a higher degree of band hybridization. Recent advancements have addressed the limitation of thermal instability of Bi contacts by employing hybrid contacts, where a Sb ($0\underline{1}12$) layer is inserted between Bi and Au layers in MoS_2_ FETs. These hybrid contacts improved on‐state current stability and long‐term device stability exceeding two months, achieving mobilities of up to 90 cm^2^ V^−1^ s^−1^ with a *R_c_* as low as 100 Ω µm (Figure 3d),^[^ ^36^ ^]^ significantly outperforming traditional Ti and Cr contacts, making them an effective contact strategy for mobility enhancement in 2D FETs.

#### 3.1.2 Van der Waals contacts

Traditional direct metal deposition methods often damage the delicate lattice structures of 2D semiconductors, introducing defects that significantly elevate *R_c_*. vdW contacts, characterized by weak vdW interactions, offer a promising alternative for creating high‐quality M–S interfaces. This approach involves transferring pre‐patterned metal electrodes onto 2D semiconductors, substantially reducing interface disorder, mitigating Fermi‐level pinning, and minimizing defect‐related gap states. Figure 3e presents a process involving the transfer of pre‐patterned metal electrodes onto MoS_2_ to form orderly stacked vdW contacts without creating interface disorder. Moreover, the gentle and low‐energy material integration strategy of vdW contacts eliminate Shockley‐Tamm states on the 2D semiconductor surface and avoids introducing defects, residues, strains, and defect‐induced gap states due to the absence of direct chemical bonding. These improvements collectively contribute to lower *R_c_*, thereby enabling higher mobility and better device performance.^[^ ^42^ ^]^ Liu et al.^[^ ^43^ ^]^ reported that vdW contacts increased the pinning factor from 0.09 to 0.96, effectively reducing *R_c_* and enhancing carrier injection efficiency, as depicted in Figure 3f.^[^ ^44^ ^]^ The positive impact of vdW contacts on MSi_2_N_4_‐based FETs showcases their potential for high‐performance 2D electronic devices. Using density functional theory (DFT), the contact types and Schottky barrier heights of MSi_2_N_4_/XY_2_ were shown to be customizable by adjusting the work function of 2D metals.^[^ ^45^ ^]^ This approach facilitated ohmic contacts with combinations like MoSi_2_N_4_/H‐NbS_2_, WSi_2_N_4_/H‐XS_2_, and WSi_2_N_4_/H‐NbSe_2_, with reduced FLP effects compared to other 2D semiconductor contacts. The FLP factors for MoSi_2_N_4_/XY_2_ (WSi_2_N_4_/XY_2_) were found to be S_N_ = 0.66 and S_P_ = 0.67 (S_N_ = 0.51 and S_P_ = 0.53), highlighting their effectiveness in engineering contacts to support high mobility operation.

Further, a universal wafer‐scale vdW metal integration strategy has also been developed, applicable to a wide range of metals and semiconductors. This method utilizes a thermally decomposable polymer as a buffer layer, allowing different metals to be deposited without damaging the underlying 2D semiconductor channels. The polymer buffer is subsequently removed through dry thermal annealing. High‐adhesion and industry‐compatible metals including Co, Ni, Al, and Ti could be integrated as contacts for 2D transistors, enabling *R_c_* tuning and improved field effect mobility.^[^ ^42^ ^]^ Larger‐scale production of vdW contacts has shown advantages in engineering mobility, exemplified by the use of chemical vapor deposition (CVD)‐grown multi‐layer graphene (MLG) sheets as flexible electrodes in WS_2_ FETs. Specifically, MLG offers superior attributes such as lower sheet resistance, enhanced mechanical durability, and tunable Fermi levels compared to single‐layer graphene, leading to reduced DOS compared to conventional metals. For instance, MLG vdW electrodes in WS_2_ transistors effectively improved mobility from 5 cm^2^ V^−1^ s^−1^ (traditional metals) to 50 cm^2^ V^−1^ s^−1^.^[^ ^46^ ^]^ This advancement highlights vdW contacts' transformative potential in large‐scale device applications, particularly benefiting bulk semiconductors previously constrained by high‐energy metallization processes and poorly defined M‐S contacts, as shown in Figure 3i.

#### 3.1.3 Edge Contact

Edge contacts strongly influence carrier mobility in 2D devices by providing superior charge injection efficiency, lower *R_c_*, and improved interface quality compared to conventional surface contacts. Edge‐contacted multilayer MoS_2_ devices consistently outperform top‐contact configurations, achieving notable mobility improvements in ultrathin samples.^[^ ^47^ ^]^ For instance, in devices with thicknesses below 5 nm, edge‐contact configurations improved mobility by two orders of magnitude due to screening from the bottom layer. On the other hand, top‐contact devices experience a 50% reduction in mobility in samples thicker than 5 nm due to interlayer resistance. Specifically, the mobility of Au top‐contact devices decreases from 120 cm^2^ V^−1^ s^−1^ at 5 nm to 5 cm^2^ V^−1^ s^−1^ at 25 nm, whereas Au edge‐contacts boosted mobility from 5 cm^2^ V^−1^ s^−1^ at 5 nm to 60 cm^2^ V^−1^ s^−1^ at 15 nm,^[^ ^48^ ^]^ highlighting the superiority of edge contacts for thicker 2D semiconductors. Moreover, edge contacts also showed the potential to reduce extrinsic scattering from sources such as charged impurities, remote optical phonons and coulomb impurity scattering. This is evident from the exceptionally high Hall mobility values achieved at low temperature: 34 000 cm^2^ V^−1^ s^−1^ for six‐layer MoS_2_ and 1020 cm^2^ V^−1^ s^−1^ for monolayer MoS_2_. In contrast, room‐temperature mobility varying between 40–120 cm^2^ V^−1^ s^−1^ across different samples were reported in other studies.^[^ ^49^ ^]^ Additionally, Wang et al.^[^ ^50^ ^]^ demonstrated near‐ballistic transport in edge‐contacted h‐BN/Graphene/h‐BN heterostructures (Figure 3g), achieving exceptional room‐temperature mobilities exceeding 140 000 cm^2^ V^−1^ s^−1^ and remarkably low sheet resistivities, approaching intrinsic graphene's theoretical acoustic‐phonon‐limited mobility and outperforming any metal at room temperature.

Despite the advantages, fabricating high‐quality edge contacts remains technically challenging, leading to variability and inconsistent device performance. Effective edge contact fabrication is highly sensitive to processing conditions, including in situ Ar‐ion sputtering and annealing treatments. Optimized edge‐contacted monolayer MoS_2_ devices have exhibited mobilities up to 30 cm^2^ V^−1^ s^−1^, about an order of magnitude higher than those of poorly prepared samples (≈3 cm^2^ V^−1^ s^−1^).^[^ ^48^, ^51^ ^]^ Notably, edge‐contact resistance shows less dependence on gate voltage than surface contacts, underscoring their potential to enhance mobility and device stability when fabrication processes are precisely controlled.^[^ ^52^ ^]^

#### 3.1.4 Non‐Destructive Contact Doping

Another emerging strategy for optimizing the M‐S interface leverages non‐destructive electrostatic doping near the contact regions to enhance mobility without compromising channel crystallinity. By applying local electric fields in the contact regions, this approach modulates carrier density at the M‐S junction, effectively lowering the Schottky barrier height and improving charge injection. As a result, *R_c_* is reduced without introducing chemical or structural perturbations while maintaining intrinsic transport properties of the 2D semiconductor channel.^[^ ^53^, ^54^, ^55^ ^]^

For instance, in MoS_2_ double‐gate FETs, local electrostatic doping of the contact regions via the bottom gate has been demonstrated to produce ohmic‐like behavior, thereby improving mobility without introducing lattice damage. Yi et al. demonstrate that the symmetric tuning of top and bottom gates could establish controlled doping gradients at the M‐S interface, achieving a sharp on/off current ratio of ≈10^7^, subthreshold swing of 65.5 mV dec^−1^, and a mobility of ≈50 cm^2^ V^−1^ s^−1^ (Figure 3h).^[^ ^54^ ^]^ Similarly, Sun et al. developed a reconfigurable WSe_2_‐based heterostructure device using a partial floating gate FET (PFGFET) design. This architecture enabled independent control of doping in the contact and channel regions, allowing for dynamic switching between *p*‐ and *n*‐type operation. The optimized carrier injection yielded an on/off ratio of 10^8^, ultralow subthreshold swing of 64 mV dec^−1^, and a mobility approaching ≈86 cm^2^ V^−1^ s^−1^.^[^ ^53^ ^]^

Such electrostatic engineering of the contacts ‐ implemented through global gates with offset doping or local gates aligned to the contact edges ‐ represents a powerful, scalable, and CMOS‐compatible strategy to complement conventional contact material or edge‐contact‐based designs. Its non‐invasive nature makes it suitable for integration into flexible electronics or vdW heterostructures, where preserving structural and electronic integrity of the channel is essential.

Collectively, these advances mentioned above underscore the critical importance of interface engineering via optimized material selection, vdW integration, edge‐contact, and non‐destructive electrostatic doping strategies in achieving high mobility and robust performance in next‐generation 2D electronic devices. *R_C_* of various contacts are summarized in Figure 3i.

### 3.2 Work Function Engineering

**Figure 4:**
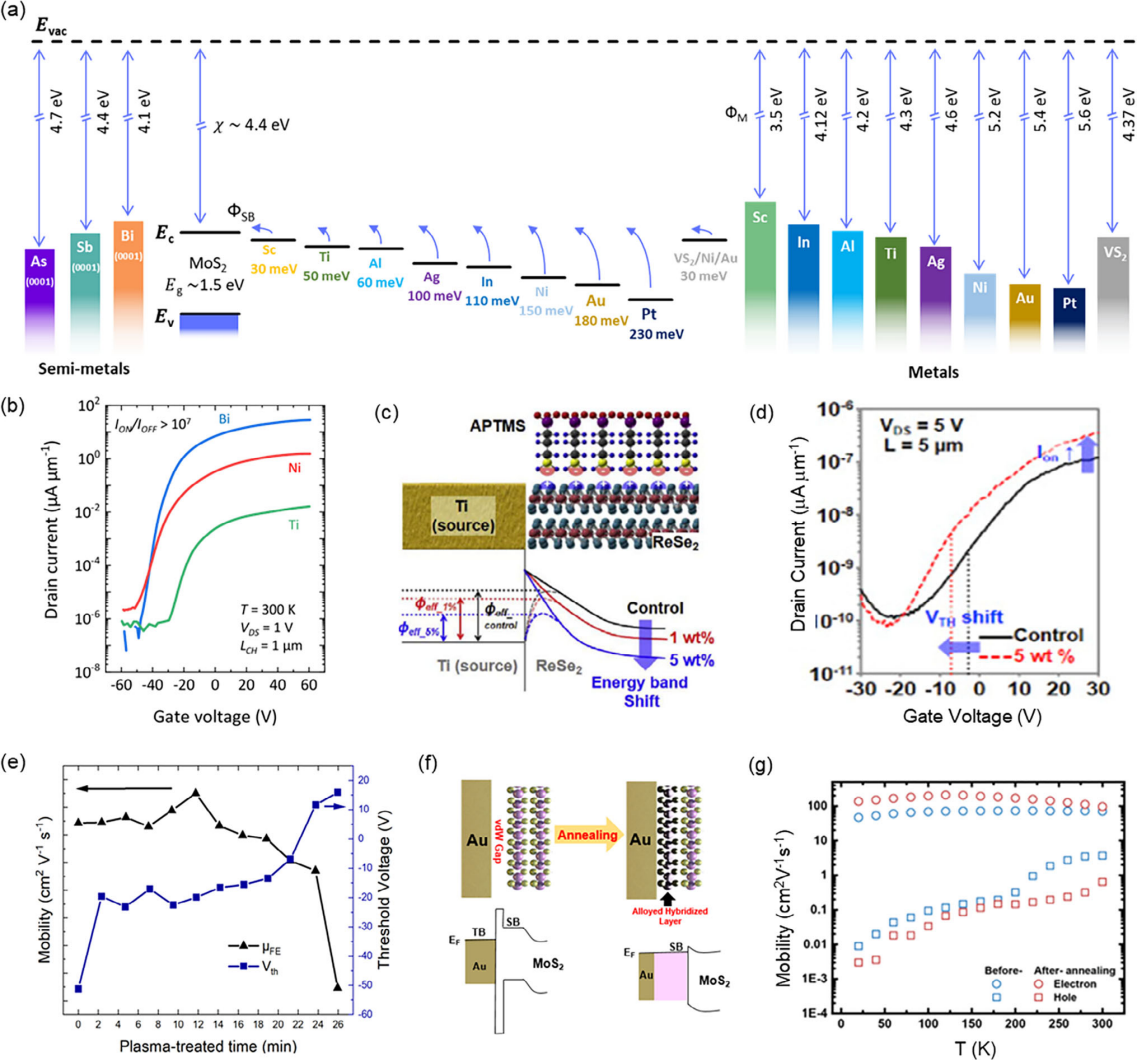
Work function engineering overview. a) Schematic diagram comparing the band alignments between metals and MoS_2_. Experimentally (middle part) obtained Schottky barrier height (Ф_SB_) differs from theoretically calculated values (left and right parts) based on metal work function (Ф_M_) and electron affinity (χ), indicating strong Fermi level pinning effect at the metal‐2D semiconductor interface.^[^ ^56^ ^]^ b) Transfer characteristics (I_DS_‐V_GS_) of monolayer MoS_2_ FETs with different contacts (Bi, Ni, and Ti) on 300‐nm‐thick SiO_2_ dielectrics are compared at room temperature. The Bi‐MoS_2_ FET exhibits a significantly I_ON_/I_OFF_ exceeding 10^7^.^[^ ^38^ ^]^ Reproduced with permission, Copyright 2021 Springer Nature. c) Schematic diagram of an APTMS‐doped ReSe_2_ photodetector and an optical image of the actual device (top) energy band diagram displays the Ti‐ReSe_2_ junction before and after APTMS doping at different weight percentages (1% and 5%) (bottom).^[^ ^61^ ^]^ d) I_D_‐V_G_ curves for both the control ReSe_2_ photodetector (solid line) and the APTMS‐doped ReSe_2_ photodetector (dotted line) under a fixed V_DS_ of 5V.^[^ ^61^ ^]^ Reproduced with permission, Copyright 2018 Elsevier B.V. e) Field effect mobility (µ_FE_), and threshold voltage (V_th_) with respect to O_2_ plasma treatment time.^[^ ^63^ ^]^ Reproduced with permission, Copyright 2017 Royal Society of Chemistry. f) Schematic illustration showcases the effects of before and after annealing on the atomic structure of MoS_2_.^[^ ^69^ ^]^ Reproduced with permission, Copyright 2020 Wiley‐VCH GmbH. g) Mobility enhancement in PdSe_2_ flakes due to annealing, indicated by red and blue shaded areas.^[^ ^70^ ^]^ Reproduced with permission, Copyright 2020 Wiley‐VCH GmbH.

Beyond interface optimization, adjusting the metal–semiconductor energy alignment through work function engineering offers another route to reduce contact resistance and improve carrier injection. The work function of a material, defined as the energy difference between its vacuum and Fermi levels,^[^ ^56^ ^]^ fundamentally dictates energy‐band alignment at M–S interfaces, critically impacting the contact resistance (*R_c_*) and thus device performance in semiconductor technologies. Specifically, the alignment of energy bands at these interfaces determines the Schottky barrier height (SBH), influencing carrier injection efficiency. Mathematically, the SBH for electron and hole injection can be expressed as: ^[^ ^44^ ^]^

(4) $$\Phi_{SB-n}=\Phi_{M}-\chi ,\Phi_{SB-p}=E_{g}+\chi -\Phi_{M}$$

where $\Phi_{SB-n}$ and $\Phi_{SB-p}$ represent the SBH for electron and hole injection, respectively. $\Phi_{M}$ represent the work function for metal, *E_g_* is the semiconductor band gap and χ is the electron affinity, which is defined as the energy difference between the conduction band (*E_c_*) and the vacuum level (*E_vac_*). The $\Phi_{SB}$ is a critical factor in determining carrier injection efficiency at the M‐S interface, directly influencing charge transport and device performance.^[^ ^56^, ^57^ ^]^ When the $\Phi_{M}$ aligns near the Fermi level (*E_F_*) within the conduction band, electron injection is facilitated. Conversely, alignment near the valence band promotes hole injection. **Figure** **4**a illustrates the band alignments of various metal‐MoS_2_ junctions alongside their respective $\Phi_{M}$.

In an ideal metal‐2D semiconductor interface, the SBH follows the Schottky–Mott rule which predicts a linear dependence of the SBH on the $\Phi_{M}$ with a slope of unity.^[^ ^58^ ^]^ However, achieving an ideal M‐S contact in practice is rare, and the estimated $\Phi_{SB}$ often deviates from the Schottky‐Mott model due to interface states and other non‐idealities, a discrepancy first observed by Bardeen in 1947.^[^ ^59^ ^]^ Therefore, accurate estimation and understanding of SBH in M–S junctions are essential for optimizing carrier injection and device performance.

#### 3.2.1 Selection of Contact Metals

Selecting appropriate contact metals is crucial to achieving optimal device performance. The $\Phi_{M}$ of various metals including Sc ($\Phi_{M}$ = 3.5 eV), Ti ($\Phi_{M}$ = 4.3 eV), Ni ($\Phi_{M}$ = 5.3 eV), and Pt ($\Phi_{M}$ = 5.6 eV) greatly affects device performance of MoS_2_, revealing a systematic decrease in the on‐state current from Sc to Pt contacts which reflects metal‐specific carrier injection efficiencies (Figure 4a).^[^ ^57^ ^]^ Despite variations from $\Phi_{M}$, MoS_2_ devices displayed *n*‐type conduction due to pronounced Fermi level pinning effects, where the metal's Fermi level remains near the conduction band edge of MoS_2_, thereby suppressing carrier injection and reducing mobility. Semi‐metals (such as Bi, Sb, As) were recently demonstrated to display ohmic contacts owing to their low work function and compatibility with the conduction band minimum of MoS_2_. Figure 4b presents the transfer curves of monolayer MoS_2_ transistors with Bi‐, Ni‐, and Ti‐contacts.^[^ ^38^ ^]^ Specifically, monolayer MoS_2_ devices employing Bi contacts exhibited exceptional electron mobility, surpassing those of traditional metals by three orders of magnitude (30 cm^2^ V^−1^ s^−1^ for Bi and 0.03 cm^2^ V^−1^ s^−1^ for Ti), highlighting the critical role of metal work function in enhancing device performance. This substantial enhancement in carrier mobility highlights the critical role of contact metal selection in high‐performance 2D electronics.

#### 3.2.2 Surface Functionalization

Surface functionalization provides an effective pathway for precise manipulation of the electronic properties of 2D semiconductors through targeted chemical modifications, thereby enabling tunable control over work functions, band alignments, and charge transport characteristics, thereby offering a powerful tool for optimizing device performance.^[^ ^60^ ^]^ Chemical functionalization methods, such as molecular adsorption or covalent attachment, offer practical strategies for overcoming interfacial limitations. For example, Ali et al.^[^ ^61^ ^]^ employed (3‐aminopropyl) trimethoxy silane (APTMS) for *n*‐doping to enhance the performance of ReSe_2_ photodetectors (Figure 4c). The amine functional groups (‐NH_2_) in APTMS played a crucial role in attracting holes and increasing the electron carrier density at the APTMS/ReSe_2_ interface, resulting in improved device characteristics. This modification reduces the effective barrier height at the Ti‐ReSe_2_ junction, resulting in a negative shift in the threshold voltage and a three‐fold increase in the on‐current ratio (Figure 4d).

Similarly, plasma‐assisted functionalization strategies, such as oxygen, nitrogen, or UV/ozone plasma treatments, have also proven highly effectiveness in enhancing device performance. Oxygen plasma treatments, for instance, can oxidize the surface layer of 2D semiconductors, increasing defect concentration and causing lattice distortion as oxygen ions bond to vacancy sites, filling localized states.^[^ ^62^ ^]^ This process can improve mobility and increase the work function of 2D TMDCs. For example, Zhang et al.^[^ ^62^ ^]^ observed an increase in MoS_2_ film thickness from 0.9 to 1.79 nm after oxygen plasma treatment, with XPS analysis showing the disappearance of Mo^4+^ peaks at 233.2 eV and 230 eV, and the emergence of Mo^6+^ peaks at 232.5 eV and 235.65 eV, indicating the transformation from MoS_2_ to MoO_3_. Kang et al.^[^ ^63^ ^]^ conducted a detailed study on HfSe_2_ FETs, optimizing layer thickness and utilizing oxygen plasma treatment which increased the current on/off ratio by four orders of magnitude, and improved field‐effect mobility by up to 40 % (Figure 4e). Nan et al.^[^ ^64^ ^]^ investigated structural defects in MoS_2_ and found that mild oxygen plasma treatment improved carrier mobility by up to 500%, increasing from 0.4‐7.8 to 11.2‐39 cm^2^ V^−1^ s^−1^. Similarly, Jiang et al.^[^ ^65^ ^]^ achieved a six‐fold enhancement in mobility in WS_2_ FETs, from 29.7 to 184.2 cm^2^ V^−1^ s^−1^, along with an increase in on/off current ratio from 10^3^ to 10^6^ using nitrogen plasma treatment. Guo et al.^[^ ^66^ ^]^ showed that UV/ozone plasma treatment enhanced MoS_2_ mobility from 2.8 to 27.6 cm^2^ V^−1^ s^−1^, thereby improving overall device performance. These results further highlight plasma‐based surface engineering as an efficient method for precise and stable work‐function tuning and performance optimization.

#### 3.2.3 Thermal Annealing

Thermal annealing offers another robust approach to modifying and optimizing M–S interfaces, enabling precise control over interface properties and the elimination of defects and impurities (Figure 4f). Controlled annealing treatments induce structural and chemical reconfigurations at M‐S interfaces, thereby directly influencing charge transport properties and improving carrier injection efficiency.^[^ ^67^ ^]^ Annealing of Au‐MoS_2_ interfaces, for example, has been reported to greatly reduce the tunnelling barrier and Schottky barrier due to improved vdW interactions and alloy formation at the interface, directly enhancing electron mobility from 8.5 to 20.7 cm^2^ V^−1^ s^−1^ upon annealing at 200 °C in nitrogen for 2 h.^[^ ^68^ ^]^ In another example, vacuum annealing at 250 °C for 1 h under vacuum led to an 80‐fold increase in mobility, from 0.1 to 8 cm^2^ V^−1^ s^−1^.^[^ ^69^ ^]^

Similarly, annealing PdSe_2_ devices at 480 K (Figure 4g) led to nearly three‐fold improvements in electron mobility (from 75 to 210 cm^2^ V^−1^ s^−1^), attributed primarily to the removal of surface contaminants, impurities, and a reduction in carrier scattering. These experimental observations consistently demonstrate that optimized annealing conditions can yield substantial improvements in mobility and overall device performance. Furthermore, the careful selection of annealing environments (such as vacuum or inert gases) and temperatures is crucial to achieving stable, reproducible results, underlining thermal annealing as a pivotal technique for interface engineering in advanced 2D electronic applications.

### 3.3 Doping Engineering

In addition to external interface modifications, directly altering the carrier concentration through doping provides a fundamental route to improve 2D device performance. Doping engineering is essential for modulating carrier density in 2D semiconductors, analogous to thermal diffusion or ion implantation methods in conventional silicon‐based technologies. However, the atomic‐scale thickness of 2D materials imposes stringent requirements on doping techniques, necessitating approaches compatible with CMOS processes that avoid structural damage to the fragile lattice. To address these challenges, various doping strategies ‐ including substitutional doping, ion implantation, and surface charge transfer ‐ have been extensively explored for TMDCs. This section critically reviews these doping methodologies, highlighting recent advancements and their impact on carrier mobility and device performance.

#### 3.3.1 Substitutional Doping

Substitutional doping involves the deliberate replacement of host atoms within the 2D lattice structure by dopant atoms, introducing donor or acceptor states to achieve *n*‐type or *p*‐type doping. Appropriate dopant selection is critical, influencing doping polarity and resultant device characteristics.^[^ ^71^ ^]^

In multilayer MoS_2_, polyethyleneimine (PEI) doping has demonstrated reduced sheet resistance and contact resistance, increasing field‐effect mobility from 20.4 to 32.7 cm^2^ V^−1^ s^−1^.^[^ ^72^ ^]^ Similarly, chemical doping of monolayer WSe_2_ with chloroauric acid (HAuCl_4_) achieved effective p‐type doping, lowering contact resistance to 0.7 kΩ · µm and increasing hole mobility from 108 to 120 cm^2^ V^−1^ s^−1^ at high doping levels of 1.76 × 10^13^ cm^−2^.^[^ ^73^ ^]^ Such enhancements can be attributed to efficient extrinsic carrier injection facilitated by chemical doping. Remote doping strategies, such as the use of defective SiO_x_ dielectric caps has achieved robust doping densities up to 1.4 × 10^13^ cm^−2^ and mobility increments from 25 to 44 cm^2^ V^−1^ s^−1^ with long‐term stability for over a month^.[^ ^74^ ^]^ Jang et al. further demonstrated solution‐deposited benzyl viologen as a remote dopant for MoS_2_, increasing mobilities from 124 to 233 cm^2^ V^−1^ s^−1^ at room temperature.^[^ ^75^ ^]^

**Figure 5:**
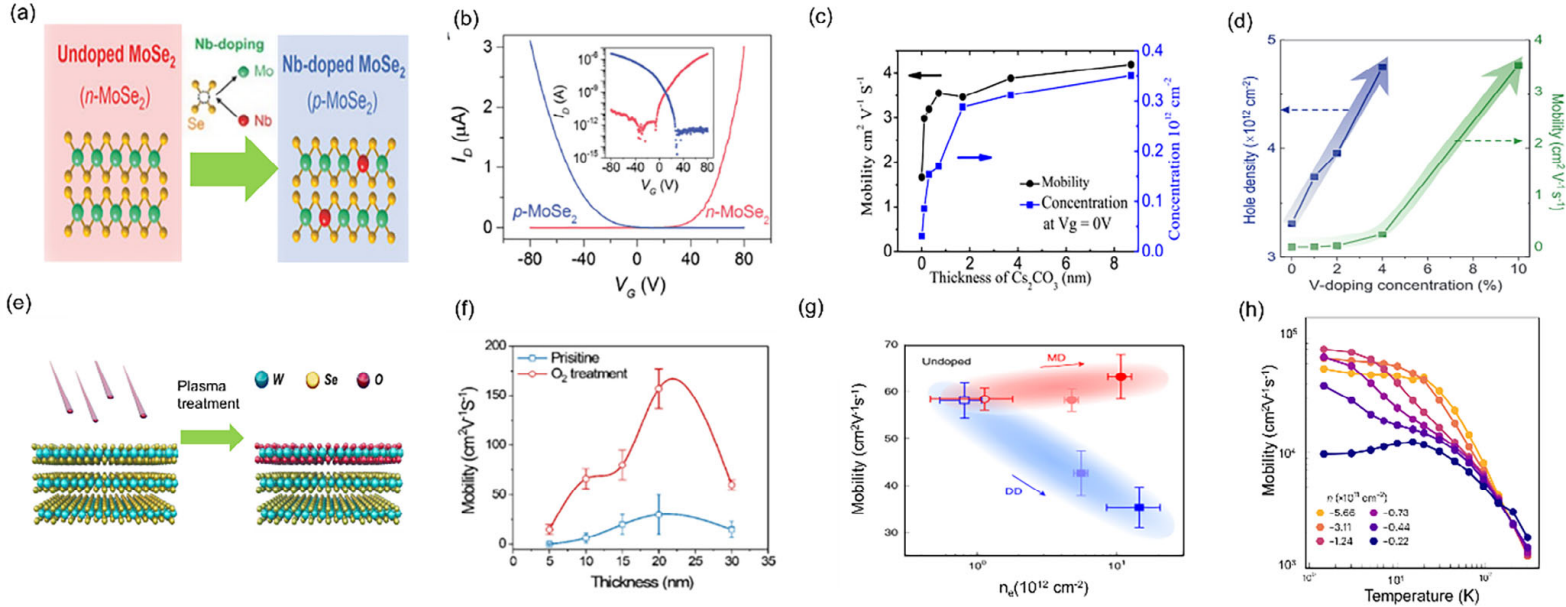
a) Vertical stacked homojunction p‐n diode in MoSe_2_ is illustrated, highlighting the Nb‐doping mechanism for p‐type MoSe_2_, where Nb atoms replace Mo atoms between Se atoms.^[^ ^76^ ^]^ Reproduced with permission, Copyright 2015 Wiley‐VCH GmbH. b) Transfer curves (I_d_‐V_g_ plot) of both n‐type and p‐type MoSe_2_ field‐effect transistors are presented, showing their electrical characteristics.^[^ ^60^ ^]^ Reproduced with permission, Copyright 2016 Wiley‐VCH GmbH. c) Field effect mobility and electron concentration at zero gate voltage were estimated as a function of the Cs_2_CO_3_ film thickness.^[^ ^77^ ^]^ Reproduced with permission, Copyright 2014 American Chemical Society. d) Field‐effect hole mobility and intrinsic hole carrier concentration measured as a function of V doping concentration of WSe_2_.^[^ ^78^ ^]^ Reproduced with permission, Copyright 2019 Wiley‐VCH GmbH. e) Schematic diagram demonstrates the O_2_ plasma treatment of bulk WSe_2_, showcasing the process and its effects.^[^ ^85^ ^]^ Reproduced with permission, Copyright 2023 The Royal Society of Chemistry. f) Mobility values obtained from WSe_2_ field‐effect transistors of different thicknesses are compared, indicating the relationship between mobility and thickness^[^ ^85^ ^]^. Reproduced with permission, Copyright 2023 The Royal Society of Chemistry. g) Field‐effect mobility (µ) is shown as a function of the electron concentration (n_e_) for the FETs before and after doping. The light blue (light red) and blue (red) filled squares (circles) represent the DD (MD) FETs after doping once and twice.^[^ ^90^ ^]^ Reproduced with permission, Copyright 2021 Springer Nature. h) Temperature‐dependent Hall mobility measurements from 1.5K to room temperature across various channel densities.^[^ ^20^ ^]^ Reproduced with permission, Copyright 2024 Springer Nature.

Beyond surface or remote doping, substitution of transition metal or chalcogen atoms has been explored (**Figure** **5**a). Niobium (Nb) doped MoSe_2_ demonstrated p‐type doping and high on/off current ratios of ≈10^7^ (Figure 5b), although mobility enhancements remained moderate (hole: ≈3 cm^2^ V^−1^ s^−1^, electron: ≈13 cm^2^ V^−1^ s^−1^).^[^ ^76^ ^]^ Caesium carbonate (Cs_2_CO_3_) treatment was shown to dope MoS_2_ and electron mobility is improved from 1.8 to 4.3 cm^2^ V^−1^ s^−1^, attributable to reduced impurity scattering.^[^ ^77^ ^]^ Similarly, Vanadium (V) doping in WSe_2_ demonstrated controlled *p*‐type doping and increased hole mobility from 0.0006 to 3.51 cm^2^ V^−1^ s^−1^ (Figure 5d).^[^ ^78^ ^]^ Chen et al. demonstrated that substitutional dopants in CVD‐grown WS_2_ using IIIA elements (e.g., B, Ga, In) can simultaneously modulate carrier polarity and enhance optoelectronic properties through defect engineering, displaying long‐term ambient stability and mobilities reaching 8 cm^2^ V^−1^ s^−1^, confirming substitutional doping as a flexible method for precise carrier control and mobility enhancement in 2D TMDCs.^[^ ^79^, ^80^ ^]^

However, substitutional doping is challenging to control owing to native defects and limited physical space, which in‐turn act as ionized impurity centers that scatter carriers via Coulomb interactions.^[^ ^80^, ^81^ ^]^ This in turn reduces mobility, especially at higher dopant concentrations or when there is poor lattice compatibility. For instance, first‐principles calculations in single‐layer MoS_2_ revealed that intrinsically poor room temperature mobility at high charged impurity densities (≥10^12^ cm^−2^) and becomes weakly dependent on dielectric environment.^[^ ^27^ ^]^ Another consideration is the size of the substitutional dopant; if vastly different from the host lattice, can introduce large magnitudes of strain, leading to lattice distortion.^[^ ^82^, ^83^ ^]^

#### 3.3.2 Ion implantation

Ion implantation, a widely used technique in conventional bulk semiconductors, enables precise control of doping concentration but also poses relevant risks of lattice damage in atomically thin 2D materials. Despite this limitation, controlled ion implantation techniques, including ionic liquid gating and plasma doping, have demonstrated substantial mobility improvements.

Ionic liquid gating offers an excellent route to enable extreme carrier modulation via electrostatic doping in 2D FETs. For example, MoS_2_ FETs employing ionic gating exhibited enhancements to electron mobility by up to 1200 % (from 5 to 60 cm^2^ V^−1^ s^−1^ at 250 K), and further to 220 cm^2^ V^−1^ s^−1^ at 77 K due to low resistance MoS_2_/metal tunneling contacts.^[^ ^84^ ^]^ However, this method is limited by its operational temperature range (≤ 250 K), where ionic liquids become unstable at room temperature due to ion mobility, and thermal cycling can introduce strain or cracks in delicate 2D layers. Plasma‐assisted doping is another variant of ion implantation that has shown promise in achieving low contact resistance and improving mobility (Figure 5e). Yue et al.^[^ ^85^ ^]^ utilized plasma treatment to convert Schottky to ohmic contacts in WSe_2_ photodetectors, enhancing mobility from 22 to 157 cm^2^ V^−1^ s^−1^ as illustrated in Figure 5e. Lee et al.^[^ ^86^ ^]^ similarly demonstrated reported a remarkable 359‐fold mobility enhancement from 0.30 to 107.8 cm^2^ V^−1^ s^−1^ in WSe_2_ FETs by depinning the Fermi level from −0.06 to −0.36, as shown in Figure 5g, where 4‐terminal (4T) mobility remains similar for Pd and In contacts but Pd enables higher 2‐terminal (2T) mobility, demonstrating efficient carrier injection.

Despite these successes, the inherent etching effects and structural defects generated by ion implantation remain problematic where vacancies, interstitials, and amorphized regions act as strong scattering centers and recombination sites that results in mobility degradation. Although careful thermal annealing, low‐energy implantation, or use of remote plasma with encapsulation can mitigate, but not fully eliminate, these defects. Consequently, careful optimization of implantation conditions and techniques is required to balance doping efficiency with structural integrity. This challenge is particularly acute in monolayer TMDCs, where the substitution‐to‐defect ratio remains poorly understood and warrants further systematic investigation.

#### 3.3.3 Surface Charge Transfer

Surface charge transfer doping presents a highly promising non‐destructive doping approach, achieving controlled n‐ or p‐type doping without much lattice distortion. Molecules such as H_2_O, NH_3_, NO_2_, and NO can adsorb onto TMDC surfaces, altering their electronic structure via physisorption or chemisorption interactions^.[^ ^70^ ^]^ The adsorption process can be classified as either physisorption, involving weak and reversible interactions, while chemisorption is characterized by strong and irreversible chemical bonding between the adsorbate and the surface. The type of doping (*n*‐ or *p*‐type) depends on the host material's electron affinity relative to the adsorbed molecule.

For example, NO_x_, H_2_O, and O_2_ tend to be *p*‐doping species, while ethanol and NH_3_ tend to be *n*‐doping species. This controllable doping method has important applications in fields such as gas sensing and filtering.^[^ ^86^ ^]^ Moody et al.^[^ ^87^ ^]^ explored Molybdenum Oxides (MoO_X_) as a charge transfer dopant for MoS_2_, demonstrating tunability in MoS_2_ FET threshold voltages by depositing different MoO_X_ stoichiometries. However, a key drawback of physorption‐based approaches is their susceptibility to environmental fluctuations where adsorbed molecules can easily desorb in ambient air, leading to unstable doping over time. To overcome this, Kim et al.^[^ ^88^ ^]^ employed laser‐induced PH_3_ doping on MoS_2_, resulting in increased photoluminescence peak intensity and a blue shift of ≈32 meV. This approach allowed precise spatial control of doping via PH_3_ chemisorption on laser‐induced sulfur vacancies, enhancing FET performance. Likewise, Iqbal et al.^[^ ^89^ ^]^ used deep ultraviolet light with nitrogen as a dopant to boost on‐state current to 53 µA and enhance electron mobility from 7.4 to 83.9 cm^2^ V^−1^ s^−1^ in multilayer WSe_2_. These enhancements were attributed to reduced Schottky barrier and Fermi level shifts owing to charge transfer between the dopant and host material.

Despite these advantages, charge‐transfer doping can introduce localized states at the interface or surface that act as shallow traps or scattering centers, particularly when dopants interact strongly with defect sites or are spatially non‐uniform. Furthermore, the long‐term chemical stability and reproducibility of such doping approaches can be challenging, especially under thermal or electrical bias cycling. To mitigate these limitations, band‐modulated heterostructures strategies have been developed. For instance, band‐engineered WSe_2_/h‐BN/MoS_2_ stacks spatially isolate charged impurities from the conduction channel, enhancing mobility (from 35 to 60 cm^2^ V^−1^ s^−1^) and improving device stability.^[^ ^90^ ^]^ A similar approach by Liu et al. demonstrated controlled p‐type doping of WSe_2_ via selective oxygen plasma doping using a h‐BN mask, achieving impressive on/off current ratios >10^8^ and mobility up to 191 cm^2^ V^−1^ s^−1^.^[^ ^91^ ^]^

Recent developments have further expanded charge‐transfer doping through type‐III band‐aligned heterostructures. Notably, *α*‐RuCl_3_ has been employed as a vdW electron acceptor in graphene and WSe_2_ devices, yielding substantial enhancements in mobility and reductions in contact resistance.^[^ ^20^, ^92^ ^]^ Specifically, *α*‐RuCl_3_ doping in WSe_2_ achieved record‐high hole mobilities of 80 000 cm^2^ V^−1^ s^−1^ at 1.5 K and 1500 cm^2^ V^−1^ s^−1^ at room temperature (at a low hole density ≈10^11^ cm^−2^) as shown in Figure 5h.^[^ ^20^ ^]^ These remarkable improvements stem from the high work function and narrow bandgap of *α*‐RuCl_3_, enabling efficient charge transfer and reduced interface resistance.

Collectively, doping engineering in 2D TMDCs – whether through substitutional doping, ion implantation, or surface charge transfer – is a powerful yet inherently complex strategy for improving charge injection and modulating transistor characteristics. Its influence on carrier mobility is not always straightforward, as it involves a delicate interplay between carrier concentration, impurity scattering, defect formation, and lattice integrity. Therefore, achieving optimal mobility requires a holistic consideration of these trade‐offs. For readers seeking deeper insights into the mechanisms and subtleties of doping in 2D materials, several comprehensive review articles are available.^[^ ^81^, ^82^, ^83^, ^93^ ^]^

### 3.4 Effective Mass

Beyond carrier concentration, the alteration of electronic band structure through effective mass engineering provides an additional strategy to improve mobility. The effective mass of charge carriers (m*) in semiconductors is a fundamental parameter that directly impacts their mobility. m* represents how electrons or holes respond to external forces within the periodic potential of a crystal lattice and is defined from the curvature of the electronic bands:

(5) $$\frac{1}{m^{*}}=\frac{1}{\hbar^{2}}\frac{d^{2}E}{dk^{2}}$$

where, ℏ is the reduced Planck constant, 𝑬 is the energy of the charge carrier, and 𝒌 is the wave vector. The second derivative ($\frac{d^{2}E}{dk^{2}}$) indicates the energy dispersion relation, reflecting the band structure's influence on carrier behavior.

**Figure 6:**
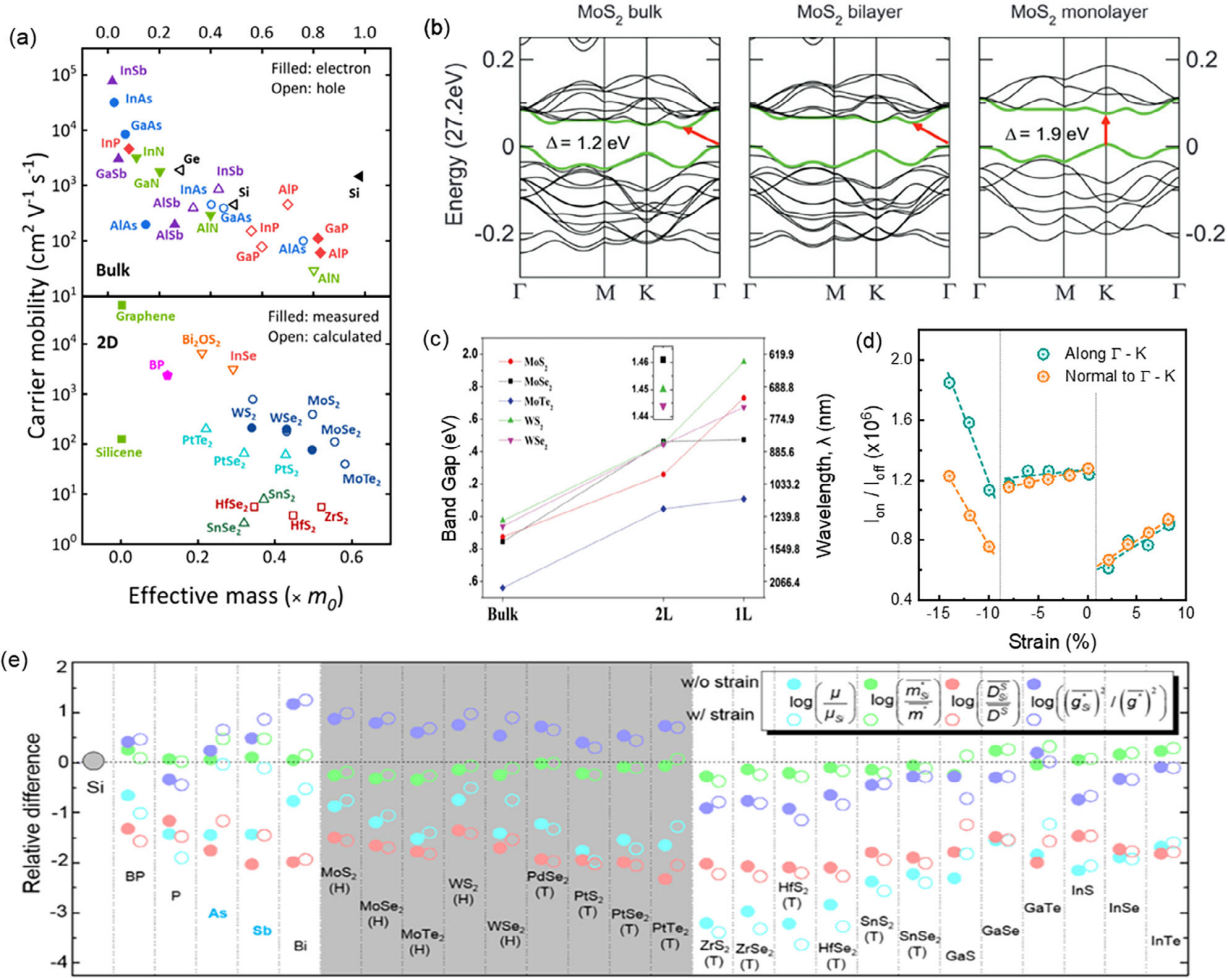
Overview of the concept effective mass a) Relationship of mobility versus effective mass across both bulk (3D) and 2D semiconductor.^[^ ^21^ ^]^ Reproduced with permission, Copyright 2021 Springer Nature. b) Band structures of MoS_2_ in bulk, bilayer, and monolayer forms. The highlighted green regions indicate the top of the valence band and the bottom of the conduction band. The red arrows mark the smallest bandgap value for each layer thickness.^[^ ^96^ ^]^ Reproduced with permission, Copyright 2012 American Physical Society. c) Relationship between the thickness of 2H‐MX_2_ materials (where M is Mo or W and X is S, Se, or Te) and their corresponding band gap energies.^[^ ^96^ ^]^ Reproduced with permission, Copyright 2012 American Physical Society. d) I_on_/I_off_ ratio of the intrinsic device, with holes as the majority charge carriers, is studied under different biaxial strains.^[^ ^100^ ^]^ Reproduced with permission, Copyright 2013 AIP Publishing. e) The differences in mobility (µ), average effective mass (m*), average effective density of scatterings (DS), and average effective electron‐phonon coupling strength (g*) between 2D semiconductors and Si. Results for materials without strain are represented by solid circles, while those with a 3% uniform tensile strain are represented by hollow circles.^[^ ^25^ ^]^ Reproduced with permission, Copyright 2020 American Physical Society.

In typical semiconductors, a lighter (heavier) m* generally yields a higher (lower) mobility (µ) through the relation:

(6) $$\mu =\frac{\left|e\right|\tau }{m^{*}}$$

with *τ* and *e* representing the average scattering time and electron charge, respectively.^[^ ^74^ ^]^ Under an applied electric field, carriers with lower m*accelerate more quickly as they experience less resistance from the lattice. In 2D semiconductors, however, additional factors (such as material thickness, mechanical strain, and surface or interface effects) introduce complexities that can influence electronic band structures to alter m* and τ (**Figure** **6**a).^[^ ^21^, ^24^ ^]^ Therefore, effective mass engineering is non‐trivial and involves careful selection of materials depending on the intended applications. In this section, two strategies commonly employed for effective mass engineering are discussed: modulating layer thickness and applying global strain.

#### 3.4.1 Layer thickness

Layer thickness governs the strength of the out‐of‐plane quantum confinement in a 2D material, reshaping band edge curvatures which alters m* (Equation 5).^[^ ^94^ ^]^ Taking MoS_2_ as an example, when thinned from bulk to a monolayer, the interlayer coupling at Γ is quenched and the bandgap migrates to the K valley, which steepens dispersion and results in a transition from an indirect to a direct bandgap (Figure 6b). First‐principles calculations by Kuc et al. reported a ≈12% reduction in the m* of MoS_2_ from 0.551*m_e_* (bulk) to 0.483*m_e_* (monolayer), that translates into a ≈15 % higher drift velocity and thus mobility under comparable scattering conditions.^[^ ^95^ ^]^ Yun et al. generalized this behavior across MX_2_ (X = S, Se, Te) materials (Figure 6c), showing that an indirect‐to‐direct bandgap transition from bulk to a monolayer exhibits a systematic decrease in m*.^[^ ^96^ ^]^ These trends are summarized in **Table** **2**.

**Table 2:** Effective masses of holes and electrons have been calculated at the high symmetry points. Values are given in units of the electron mass (m_e_).^[^ ^22^, ^126^, ^127^, ^128^ ^]^ Note that effective masses across different studies may differ depending on the assumptions employed during calculations.

Material	Carrier type	Symmetry point	Bulk	2L	1L	Refs.
MoS_2_	Hole	Γ	0.711	1.168	3.524	[22]
		K	0.625	0.628	0.637	[22]
	Electron	Midpoint of Γ–K	0.551	0.579	0.569	[22]
		K	0.821	0.542	0.483	[22]
MoS_2_	Hole	Γ	0.838	1.039	0.543	[126]
		K	–	0.548	0.546	[126]
	Electron	Midpoint of Γ–K	–	–	0.506	[126]
		K	0.590	0.521	0.504	[126]
MoS_2_	Hole	Γ	–	0.459	0.455	[128]
		K	–	0.490	0.428	[128]
	Electron	Midpoint of Γ–K	–	0.386	0.342	[128]
		K	–	0.390	0.350	[128]
MoSe_2_	Hole	Γ	0.973	1.430	0.578	[126]
		K	–	0.595	0.588	[126]
	Electron	Midpoint of Γ–K	0.521	0.539	0.502	[126]
		K	0.776	0.539	0.503	[126]
WS_2_	Hole	Γ	0.832	1.239	0.339	[126]
		K		0.345	0.339	[126]
	Electron	Midpoint of Γ–K	0.569	0.359	0.349	[126]
		K	0.665	0.359	0.347	[126]
WSe_2_	Hole	Γ	0.997	1.322	0.341	[126]
		K		0.349	0.348	[126]
	Electron	Midpoint of Γ–K	–	0.411	0.345	[126]
		K	0.489	0.412	0.345	[126]
BP	Hole	Γ	6.35	–	–	[127]
		K	0.15	–	–	[127]
	Electron	Midpoint of Γ–K	1.12	–	–	[127]
		K	0.17	–	–	[127]

Tuning of layer thickness is equally powerful in lesser studied materials such as PdSe_2_, where increasing the thickness from a monolayer to eight layers enlarges the conduction band valley‐splitting from 30 to 56 meV while lowering m* by up to 70 %, which pushes the mobility peak upward from 75 to 210 cm^2^ V^−1^ s^−1^.^[^ ^70^ ^]^

In the simple Drude picture (Equation 6), a lighter m* translates linearly into a higher mobility if the scattering time (τ) remains constant. In reality, however, scattering physics evolves with thickness. Bilayer MoS_2_, for instance, is reported to outperform its monolayer counterpart (Hall mobilities of ≈100 cm^2^ V^−1^ s^−1^ vs ≈20 cm^2^ V^−1^ s^−1^ at 300 K) owing to stronger screening of charged impurities and remote phonons.^[^ ^97^ ^]^ Conversely, ultrathin PdSe_2_ benefits from enhanced valley degeneracy which boosts conductivity but suffers a lower mobility owing to impurity and phonon scattering.^[^ ^70^ ^]^ Therefore, the full potential of layer‐thickness engineering for mobility optimization is only realized when coupled with considerations on contacts and scattering physics.

#### 3.4.2 Global strain

Mechanical strain offers a direct means of modifying band structure in 2D semiconductors and hence the m*. Strain has been proven beneficial for carrier mobility in transistors in traditional Si technology, where tensile strain improves electron mobility by splitting degenerate conduction band valleys, while compressive strain enhances hole mobility by lifting the degeneracy between light and heavy hole bands.^[^ ^98^ ^]^

Unlike bulk Si, atomically thin 2D semiconductors possess a large surface‐to‐thickness ratio and a low electrical permittivity (κ), thus even modest lattice deformations or strain can reshape the entire band structure to modify m* for mobility engineering. The low κ can be compensated by growing or depositing a high‐κ dielectric (SiN_x_, Al_2_O_3_, HfO_2_, etc.),^[^ ^99^ ^]^ where the choice of dielectric environment (type and thickness) determines the type of strain applied and influences carrier/defect screening.^[^ ^100^ ^]^

The effectiveness of biaxial strain on the electronic band structure of single‐layer MoS_2_ and its FET performance was reported theoretically to induce a semiconductor‐metal transition and capable of enhancing current on/off ratios by up to 400% (Figure 6d).^[^ ^100^ ^]^ Experimentally, tensile strain induced by depositing MgO_x_ stressor have demonstrated a boost to electron mobility of MoS_2_ by 23%, from 11.6 to 18.8 cm^2^ V^−1^ s^−1^, attributed to reduced m* and intervalley scattering. A linear relationship between mobility and strain is observed, with an enhancement rate of 130 ± 40 per % biaxial strain.^[^ ^101^ ^]^ Depositing a 30 nm thick HfO_2_ layer via ALD onto dual‐gated MoS_2_ devices led to enhanced electrostatic doping, gate control, and modulation of charge density and gate capacitance, resulting in a substantial increase in mobility from 18 to 184 cm^2^ V^−1^ s^−1^.^[^ ^30^, ^102^ ^]^ Mechanical lamination of MoS_2_ transistors on flat substrates introduces a uniform tensile strain of 1.36% that enhances electron mobility by 64% from 43.5 to 71.3 cm^2^ V^−1^ s^−1^, while preserving dielectric integrity.^[^ ^103^ ^]^ Strain imposed during CVD growth of 2D materials can be equally effective. By optimizing growth parameters (such as temperature, pressure, gas flow rates, and substrate selection), biaxial tensile strain of ≈1% was observed in monolayer MoS_2_, boosting mobility from ≈0.15 to 23 cm^2^ V^−1^ s^−1^.^[^ ^104^ ^]^

First‐principles calculations, backed by reformulated Boltzmann transport equations, recently revealed that strain alters not only the electronic band structure, but also the phonon distribution in 2D materials. This modifies electron‐phonon interactions and their scattering which can enhance mobility.^[^ ^25^ ^]^ Figure 6e shows strained 2D materials exhibiting a gain in mobility values.

Despite the advantages, achieving uniform strain across wafer scales during growth or after fabrication is technically challenging and requires careful processing conditions to minimize variability and inconsistencies. Taken together, global strain is a complementary route for effective mass tuning and can offer an attractive pathway for mobility engineering.

### 3.5 Scattering Engineering

Even with a favorable effective mass, carrier mobilities remain limited by scattering processes, making scattering engineering a vital component of mobility enhancement. Mobility in 2D semiconductors depends not only on the carrier effective mass (m*) but also on the scattering mean free time (τ_*m*_), as defined in Equation (6).^[^ ^105^ ^]^ Due to their atomically thin channel, charge carriers in 2D semiconductors are particularly susceptible to both intrinsic and extrinsic scattering sources. Consequently, engineering of the various scattering processes within the material becomes vital for reducing the impact of these interactions to recover the high intrinsic mobilities predicted by theory.^[^ ^106^ ^]^

Matthiessen's rule states that the overall resistivity (*ρ*) of a material is the sum of contributions from impurities and defects (*ρ_0i_*) and from temperature‐dependent phonon scattering (*ρ(T)*)^[^ ^107^ ^]^:

(7) $$\rho =\rho_{0i}+\rho \left(T\right)$$

Alternatively, Matthiessen's rule can also be expressed in relation to the scattering time or relaxation time (τ) of charge carriers in a material:

(8) $$\frac{1}{\tau }=\frac{1}{\;\tau_{i}}+\frac{1}{\;\tau_{ph}}$$

where τ*_i_* and τ_*ph*_ is the scattering time due to impurities and lattice scattering (phonons), respectively. This rule can be employed to analyse the contributions of different scattering mechanisms to the overall carrier dynamics in a material.^[^ ^108^ ^]^

**Figure 7:**
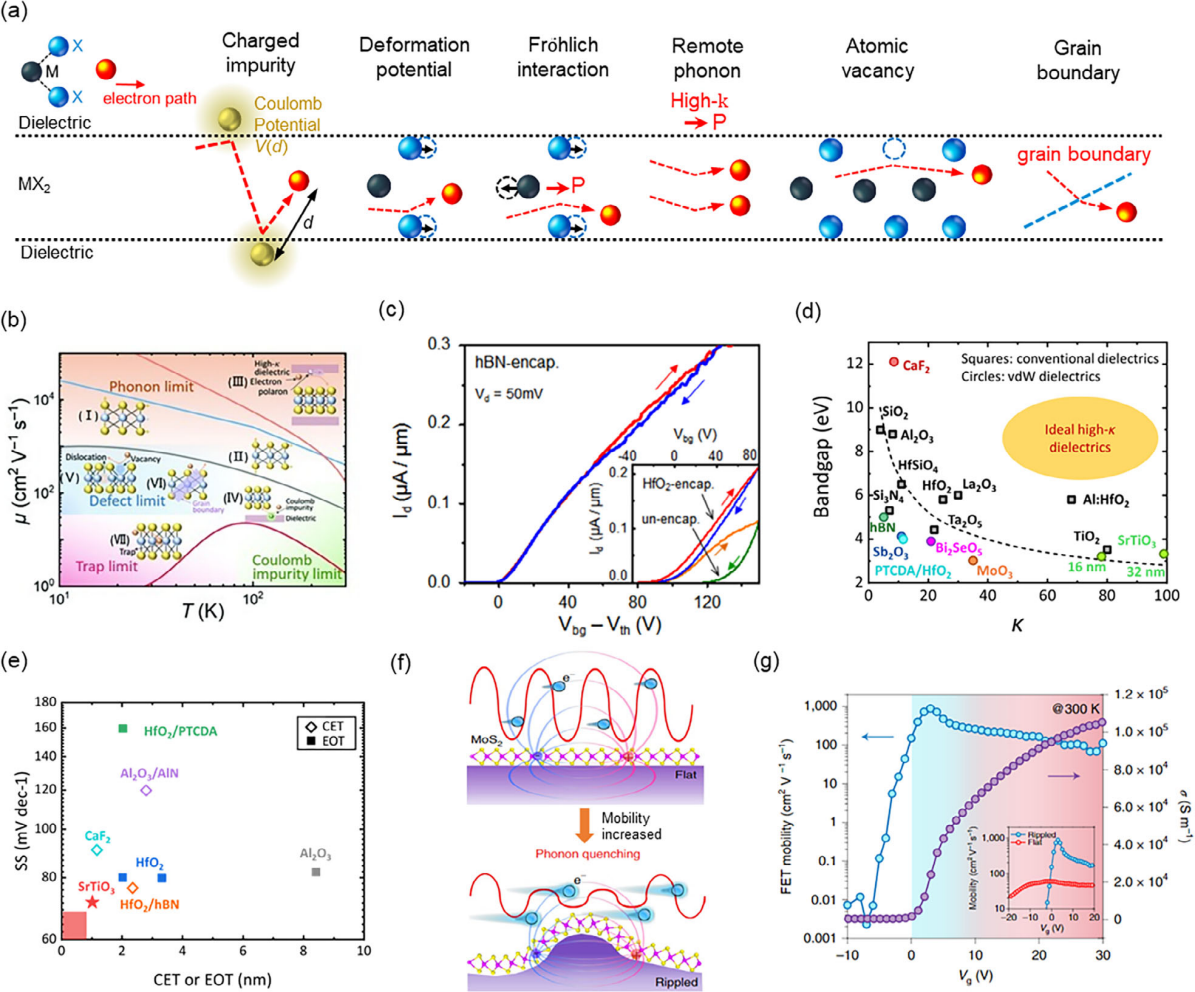
Overview of the concept Scattering Engineering a) Illustrate the various extrinsic charge carrier scattering mechanisms in a 2D TMDC/MX_2_ channel. The M and X atoms are represented by black and blue balls, respectively. The paths of electrons are shown in orange with dashed arrows indicating their trajectories. Scattering events are indicated by changes in the direction of the carrier path. Charged impurities are depicted in yellow, and their scattering potentials are represented by smeared yellow regions. The red arrow denotes the presence of polar phonons in the top dielectric.^[^ ^22^ ^]^ Reproduced with permission, Copyright 2016 The Royal Society of Chemistry. b) Schematic illustration depicting the relationship between mobility and temperature for a typical MoS_2_ transistor with various scattering mechanisms. Factors include I phonon limit, II Frohlich interaction caused by polar optical phonons, III surface phonon scattering, IV Coulomb impurity scattering, V Atomic defect, VI Defect and grain boundary VII trap effects influence the mobility trends.^[^ ^105^ ^]^ Reproduced with permission, Copyright 2023 American Association for the Advancement of Science. c) Back gated transfer curve (I_d_‐V_bg_) of an h‐BN encapsulated trilayer MoS_2_ device, exhibiting a high two terminal mobility of 69 cm^2^V^−1^s^−1^ without hysteresis in comparison. Inset: transfer curves of unencapsulated and HfO_2_ encapsulated trilayer MoS_2_ devices, shows large hysteresis and lower mobilities of 7cm^2^V^−1^s^−1^ and 18cm^2^V^−1^s^−1^, respectively.^[^ ^113^ ^]^ Reproduced with permission, Copyright 2015 American Chemical Society. d) Illustrates the correlation between the energy bandgap and the relative static permittivity for 3D oxides vdW dielectrics. Showcasing gate leakage current from typical 3D oxides and vdW dielectrics for MoS_2_ transistors with different EOT values.^[^ ^109^ ^]^ Reproduced with permission, Copyright 2022 Springer Nature. e) SS values achieved by CVD‐prepared MoS_2_ FETs with sub‐10‐nm EOT or CET technologies. The ball‐and‐stick symbol represents the EOT, where the ball represents the theoretical value and the stick represents the EOT range obtained from practical permittivity in nanocapacitors. The shaded red corner represents the low‐power specification for 2028 according IRDS.^[^ ^120^ ^]^ Reproduced with permission, Copyright 2022 Springer Nature. f) Diagram shows the comparison between f‐MoS_2_ (freestanding MoS_2_) and r‐MoS_2_ (strained MoS_2_) structures. In r‐MoS_2_, the lattice distortion induced by the substrate increases the electric polarization, resulting in an enhanced ε that improves dielectric screening and reduces E‐P scattering. This leads to a significant increase in carrier mobility. The strength of dielectric screening is illustrated by the electric‐field lines between positive and negative charges, while the red solid lines represent phonon vibrations. The pink and yellow spheres represent Mo and S atoms, respectively, and the change in electric polarization within f‐MoS_2_ and r‐MoS_2_ is depicted.^[^ ^10^ ^]^ Reproduced with permission, Copyright 2022 Springer Nature. g) Four‐probe FET mobility and electrical conductivity of r‐MoS_2_ at 300 K, varying with the back‐gate voltage. The main scattering mechanism in r‐MoS_2_ is C‐I scattering, which is dominant due to enhanced dielectric screening and suppression of E‐P scattering. The blue‐shaded region represents the linear regime just above the threshold, where the mobility reaches its peak and exhibits a quadratic dependence on dielectric screening (µ∝τ_C‐I_∝ε^2^). In the red‐shaded region, the mobility gradually decreases due to increased scattering from short‐range disorders. Inset provides a comparison of mobility as a function of gating between r‐MoS_2_ and f‐MoS_2_.^[^ ^10^ ^]^ Reproduced with permission, Copyright 2022 Springer Nature.

The manipulation of scattering dynamics of charge carriers within materials or structures can drastically tailor the electronic properties of materials. For monolayer MoS_2_, the theoretical phonon‐limited mobility at 300 K is ≈410 cm^2^ V^−1^ s^−1^, yet experiments often report much lower mobilities (ranging from 0.1–50 cm^2^ V^−1^ s^−1^) because of scattering from phonons, impurities and surface roughness.^[^ ^29^ ^]^ **Figure** **7**a illustrates the primary scattering sources, including charged impurities, deformation potential, Fröhlich interaction, remote phonons, atomic vacancies, and grain boundaries. In practice, the dominant scattering mechanism is identified by analyzing the power‐law behavior of the temperature‐dependent mobility (µ ∝ T^−γ^).^[^ ^30^, ^49^ ^]^

The interpretation of γ differs from bulk to 2D semiconductors, and this disparity stems from reduced dimensionality, weaker screening, and distinct scattering mechanisms. Figure 7b illustrates the temperature‐dependent mobility trends for MoS_2_ and the various types of scattering mechanisms conditions.^[^ ^105^ ^]^ Studies have report γ values from ‐4.58 to 3 for various 2D materials, reflecting the complex interplay of scattering mechanisms.^[^ ^10^, ^30^, ^49^, ^96^, ^110^, ^111^ ^]^ Values of γ ≈ ±1.5 generally indicate phonon‐dominated (negative) or charged‐impurity‐dominated (negative) scattering. Monolayer or few‐layer MoS_2_ on SiO_2_ typically yields γ ≈ 0.55‐1.7 at temperatures above 100 K.^[^ ^30^, ^99^ ^]^ The wide range of values in γ typically originates from extrinsic scattering and disorder (either from SiO_2_’s surface roughness and defects, or from post‐fabrication polymer residues). Although the quality of devices has improved compared to early days, 2D semiconductors are still limited by phonon scattering, resulting in poor mobility ranging from 10 to 100 cm^2^ V^−1^s^−1^.^[^ ^10^ ^]^ To address the adverse effects arising from extrinsic and intrinsic scattering sources, various methods to manipulate scattering mechanisms have been developed toward improving carrier mobility. In this section, we review commonly employed approaches, including but not limited to, encapsulating devices with hexagonal boron nitride (h‐BN), increasing dielectric screening, and local strain.

#### 3.5.1 h‐BN encapsulation

Hexagonal boron nitride (h‐BN) is widely regarded as an ideal insulator to be employed as a substrate due to its atomically flat surface, ultralow trap density, low surface roughness, and excellent dielectric properties. Numerous studies have shown that replacing SiO_2_ with h‐BN as the supporting dielectric revealed intrinsic transport‐first in graphene,^[^ ^112^ ^]^ and more recently in TMDCs. The lack of dangling bonds in h‐BN preserves carrier mobility and allows device to withstand high temperatures up to 1500 °C, making it a superior alternative to conventional 3D dielectric oxides.^[^ ^112^ ^]^ For example, h‐BN encapsulated, edge‐contacted graphene achieved room‐temperature mobility exceeding 140000 cm^2^ V^−1^ s^−1^ and a reduced sheet resistivity below 40 Ω/cm^2^ at a carrier density above 4 × 10^12^ cm−^2^, and a low R_c_ of 100 Ω·µm, comparable to the theoretical limit set by acoustic phonon scattering.^[^ ^50^ ^]^ Tri‐layer MoS_2_ demonstrated a five‐fold mobility boost from ≈ 8 to 45 cm^2^ V^−1^ s^−1^ when transferred onto h‐BN, with negligible hysteresis in transfer characteristics (Figure 7c).^[^ ^60^ ^]^ Fully encapsulated, edge‐contacted MoS_2_ pushes hall mobility can reach up to 34,000cm^2^ V^−1^ s^−1^ for 6L‐MoS_2_ and 1020cm^2^ V^−1^ s^−1^ for monolayer MoS_2_ at low temperatures due to high interface quality and reduced scattering.^[^ ^49^ ^]^

Beyond pure transport, h‐BN also possesses high impermeability that effectively shields reactive metals and serves as a protective barrier for 2D devices from environmental degradation even at elevated temperatures. A study found that h‐BN encapsulated MoS_2_ devices retain electrical performance after four months of exposure to ambient conditions and withstand temperatures of 200 °C without oxidation.^[^ ^113^ ^]^ A similar protective role is observed in WSe_2_ devices employing ionic‐liquid gating, where h‐BN encapsulation enables independent electrostatic tunability and improved contact quality, achieving high Hall mobility of >600 cm^2^ V^−1^ s^−1^ at 220 K.^[^ ^114^ ^]^ In black phosphorus, h‐BN encapsulation suppresses surface degradation and improves electron mobility by over one order of magnitude, reaching 305 cm^2^ V^−1^ s^−1^.

Although h‐BN encapsulation offers exceptional benefits, challenges remain in synthesis complexity, interface compatibility, cost, and scalability, requiring further research to fully harness the potential of h‐BN in electronic devices.^[^ ^115^ ^]^

#### 3.5.2 Dielectric Screening

Dielectric screening refers to the ability of a dielectric material to shield charge carriers from extrinsic electric fields, thereby modulating their movement and scattering. Analogous to the preceding discussion, the dielectric environment surrounding a 2D channel can introduce varying levels of screening and scattering phenomena to affect charge transport. These includes charged impurity scattering, interface roughness, and phonon interactions, where each can either result in mobility degradation or enhancement. Studies have shown that the room‐temperature mobility of most 2D semiconductors is inherently limited by a high density of scattering events (Figure 7a).^[^ ^25^ ^]^ Careful selection of dielectric environment can strategically manipulate scattering sources to modulate carrier mobility for performance optimization.

Silicon dioxide (SiO_2_) is the most commonly employed dielectric material in 2D devices, yet its low dielectric permittivity (ε_r_ ≈ 3.9) offers weak screening and leaves carriers exposed to Coulomb disorders (surface impurities and defects). Substituting SiO_2_ with high‐k dielectrics (such as Si_3_N_4_, Al_2_O_3_, HfO_2_, etc.) has been demonstrated to improve dielectric screening which attenuates the impact of Coulomb disorders on carrier mobility (Figure 7d).^[^ ^60^ ^]^ For example, Yu et al.^[^ ^26^ ^]^ compared the performance of MoS_2_ FETs across different dielectric materials (SiO_2_, Al_2_O_3_, and HfO_2_) and showed that mobility is mainly limited by charged impurity scattering at high carrier densities of ≈10^13^ cm^−2^. Room temperature mobility of monolayer MoS_2_ on HfO_2_ (ε_r_ = 16.5) reached 148 cm^2^ V^−1^ s^−1^, which is 85 % and 31 % higher than on SiO_2_ (ε_r_ = 3.9, µ = 80 cm^2^ V^−1^s^−1^) and on Al_2_O_3_ (ε_r_ = 10, µ = 113 cm^2^ V^−1^s^−1^), respectively. High quality HfO_2_ also reduces surface roughness, interface states, and leakage, enabling an effective oxide thickness (EOT) of 1 nm, impressive on/off ratios exceeding 10^7^ and remarkably low subthreshold swings of 60 mV/dec in CVD MoS_2_.^[^ ^116^ ^]^ Plasma‐treated atomic layer deposition (ALD) Al_2_O_3_ films were shown to passivate oxygen vacancies, enhancing carrier mobility of MoS_2_ from 2.4 to 39.3 cm^2^ V^−1^s^−1^ and lowering subthreshold swing to 90 mV dec^−1^.^[^ ^117^ ^]^ In a more recent study, UV‐assisted ALD Al_2_O_3_ on graphene doubled hole mobility to 1221 cm^2^ V^−1^ s^−1^, attributed to denser Al_2_O_3_ films and improved interface quality.^[^ ^118^ ^]^

Beyond conventional oxides, vdW dielectrics such as calcium fluoride (CaF_2_) and Strontium‐Titanium‐Oxide (SrTiO_3_) have emerged as effective gating materials (Figure 7e). Epitaxial CaF_2_ forms a quasi vdW interface with 2D semiconductors, delivering low leakage currents (<10^−8^ A), subthreshold swings down to 90mVdec^−1^, on/off ratios up to 10^7^, and small hysteresis.^[^ ^119^ ^]^ Free‐standing SrTiO_3_ membranes showed similar characteristics with steeper subthreshold swing of 70 mV dec^−1^, but coupling of lattice vibrations from metal‐oxide bonds with carriers can result in low carrier mobility of 39.7 cm^2^ V^−1^ s^−1^.^[^ ^120^ ^]^

Recent advancements have explored a complementary strategy exploiting native‐oxide dielectrics grown from the 2D semiconductor itself. Controlled oxidation of Bi_2_O_2_Se yields an atomically thin high‐k dielectric of Bi_2_SeO_5_ (ε_r_ = 21, EOT = 0.9) with a sharp and defect‐free interface. The resulting FETs exhibit carrier mobilities exceeding 300 cm^2^ V^−1^ s^−1^, current on/off ratios >10^5^, and a subthreshold swing of ≈75 mV dec^−1^ while maintaining low gate leakage currents comparable to that of conventional SiO_2_.^[^ ^121^ ^]^ These results highlight how lattice‐matched, high‐k dielectrics can simultaneously screen Coulomb scattering and preserve interface quality for driving advancements in 2D semiconductor‐based devices.

#### 3.5.3 Local Strain

Local strain engineering involves applying a localized mechanical stress or strain to a material to alter electronic properties by modulation of its band structure, effective mass, and carrier scattering rates. In the case of 2D materials, local strain can be induced by creating lattice distortions (such as crest, bulges, ripples, wrinkles, bubbles).

Contrary to the conventional belief that smooth and flat films are essential for achieving optimal charge transport in 2D materials, recent studies have shown that the deliberate introduction of local strain can modify electronic band structure and carrier dynamics, enabling improved mobility, tunable bandgaps, and enhanced charge injection. Pioneering studies revealed that intrinsic ripples in freestanding graphene led to a nearly 10‐ fold increase in carrier mobility.^[^ ^122^ ^]^ Similarly, it was found that employing crested substrates for MoS_2_ transistors led to a 82‐fold increase in carrier mobility from 10 to 820 cm^2^ V^−1^ s^−1^, accompanied with outstanding high saturation currents as compared to standard devices.^[^ ^123^ ^]^ Most notably, the implementation of bulged substrates to induce ripples in MoS_2_ has set a new benchmark for room‐temperature mobility surging by two orders of magnitude to ≈900 cm^2^ V^−1^ s^−1^, surpassing predicted values for flat MoS_2_ (Figure 7g). These ripples induced local lattice distortions that enhances dielectric constant in MoS_2_ and alters the distribution of phonon density of states, ultimately suppressing the effective density of electron‐phonon scattering.^[^ ^10^ ^]^

These findings collectively underscore the transformative potential of local strain engineering using substrate design for unlocking the full potential of 2D semiconductors, opening new avenues for performance optimization.

## 4 Perspective and Conclusion

The rapid evolution of 2D materials holds immense potential for continued device scaling and as complementary technologies to enhance and extend the capabilities of traditional silicon‐based systems. This review has highlighted the critical role of mobility engineering in unlocking the full potential of 2D semiconductors, spanning from materials‐level advances to device optimization and ultimately system‐level integration.

Mobility degradation‐primarily due to phonon scattering within the channel and high contact resistance at metal–semiconductor interfaces‐remains a central obstacle. To address this, strategies such as scattering engineering, effective mass engineering, doping optimization, and interface engineering have been developed. These approaches reduce detrimental phonon interactions, optimize band structures, and improve contact injection efficiency, enabling room‐temperature mobilities to rise from a few cm^2^ V^−1^ s^−1^ to over 1000 cm^2^ V^−1^ s^−1^ in various 2D semiconductors. Such improvements not only enhance individual device performance but also lay the foundation for their integration into increasingly complex electronic systems.

Despite these advancements, translating laboratory‐scale breakthroughs into industry‐ready technologies remains a challenge. Key hurdles include the synthesis of wafer‐scale, uniform, and defect‐free 2D films, precise doping control without lattice damage, the reproducibility and scalability of contact engineering techniques such as vdW and edge contacts, and the suppression of detrimental scattering mechanisms that continue to limit carrier mobility. Furthermore, balancing mobility enhancement with fabrication complexity and cost remains essential for practical integration into commercial electronics and compatibility with CMOS processes.

Ultimately, the future of 2D FETs depends on coordinated progress across materials synthesis, device engineering, and system integration. Their intrinsic advantages‐high carrier mobility and low power consumption‐make them promising candidates for dense integration in next‐generation computing architectures. Overcoming current challenges at each level will synergistically enable the creation of powerful, energy‐efficient, and scalable electronic systems. As a specialized complement to silicon, 2D technologies are poised to drive innovations in electronic design that go beyond the capabilities of conventional silicon‐based systems.
